# Supplementary material for: The structure of a hibernating ribosome in a Lyme disease pathogen
Source: Nat Commun. 2023 Oct 31;14:6961. doi: 10.1038/s41467-023-42266-7 (PMC10618245; doi:10.1038/s41467-023-42266-7)
Supplement: Supplementary file 1 — Supplementary Information [file 41467_2023_42266_MOESM1_ESM.pdf]

Supplementary Information for:

The structure of a hibernating ribosome in a Lyme disease pathogen

Manjuli R. Sharma[1], Swati R. Manjari[1], Ekansh K. Agrawal[1,a], Pooja Keshavan[1,b], Ravi K. Koripella[1,c], Soneya Majumdar[1], Ashley L. Marcinkiewicz[2], Yi-Pin Lin[2,3], Rajendra K. Agrawal[1,3,*], and Nilesh K. Banavali[1,3,*]

1. Division of Translational Medicine, Wadsworth Center, New York State Department of Health, Albany, NY

2. Division of Infectious Diseases, Wadsworth Center, New York State Department of Health, Albany, NY

3. Department of Biomedical Sciences, School of Public Health, University at Albany, Albany, NY

a. Present address – University of California at Berkeley, Berkeley, CA

b. Present address – Robert P. Apkarian Integrated Electron Microscopy Core, Emory University, Atlanta, GA

c. Present address – National Center for Biological Sciences, Bangalore, India

*nilesh.banavali@health.ny.gov, rajendra.agrawal@health.ny.gov

Supplementary Note 1. Finding the bS22 ribosomal protein in *Borrelia (Borreliella) burgdorferi* (*Bbu*).

A small helical density in our *Bbu* 70S ribosome cryo-EM map corresponded to the bS22 protein seen in mycobacterial[1-4] and Bacteroidetes[5] ribosomal small subunit structures, indicating that the bS22 protein is also present in *Bbu*. Since this protein is not annotated in the *Bbu* genome, the mycobacterial bS22 sequence from *Mycobacterium smegmatis* (*Msm*): MGSVIKKRRKRMSKKKHRKLLRRTRVQRRKLGK; was used as a query sequence for a tblastn[6] search of the *Bbu* genome with a word size of 2 and no low complexity filter. This yielded hits for a smaller fragment of the query sequence but no plausible *Bbu* bS22 protein with a size near 30 amino acid (aa) residues upon translation of the genomic hits in 6 frames. The same search was then attempted with the Bacteroidetes bS22 sequence from *Flavobacterium johnsoniae* (*Fjo*): MPSGKKRKRHKVATHKRKKRARANRHKKKK. This search yielded one hit, which when translated in the 6 frames, yielded the plausible bS22 sequence: VPCGRKRKLKKISTHKRKKKRRKNRHKKKNK-, with an appropriately placed stop codon "-". The first valine residue is likely translated as a methionine residue since substitution for Met (AUG) instead of Val (GUG) can occur due to the anticodon for fMet-tRNA (CAU) pairing well enough with GUG on the mRNA through wobble pairing between G and U. Such usage of GUG as a start codon in bacteria is known to occur at an average of 12%[7,8]. This sequence fits well into the bS22 sidechain cryo-EM densities, allowing it to be identified as the correct *Bbu* bS22 sequence.

A ClustalW 2.1[9] pair-wise sequence alignment between *Bbu* and *Fjo* bS22 sequences shows a 65% sequence identity, which explains why the *Fjo* query sequence tblastn search was successful:

```
Bbu          MPCGRKRKLKKISTHKRKKKRRKNRHKKKNK
Fjo          MPSGKKRKRHKVATHKRKKRARANRHKKKK-
             **.*:*** :*::******: * ******:
```

A ClustalW 2.1[9] multiple sequence alignment between *Bbu*, *Fjo*, and *Msm* bS22 sequences shows that identity of residues between the three sequences drops to 19%, which explains why the *Msm* query sequence tblastn search was unsuccessful:

```
Bbu          MPCGRKRKLKKISTHKRKKKRRKNRHKKKNK--
Fjo          MPSGKKRKRHKVATHKRKKRARANRHKKKK---
Msm          MGSVIKKRRKRMSKKKHRKLLRRTRVQRRKLGK
             *  .  *.. :.::.:*..*  * .* :..:
```

The genomic sequence identified for the *Bbu* B31 bS22 protein (Genbank ID: AE000783.1, nucleotides 867605-867697, *bb0822*) is:

GTGCCTTGCGGAAGAAAAAGAAAATTAAAAAAAATTTCTACCCATAAAAGGAAGAAAAAAAGAA GAAAAAATAGACATAAGAAAAAAAATAAG

Supplementary Note 2. Alignments of proteins that sequester Anti-Shine Dalgarno (ASD) sequence.

<u>bS6, Identity: 23/141 (16.3%), Similarity: 50/141 (35.5%)</u>

```
    1        10          20        30        40       50        60
Bbu MIKRYEACFLFKS--EEIEYKGSLEEVKKSLEFFGATDVVSNFIGERALEYPIKKQARGRYEIIEFS
Fjo -MNHYETVFILNPVLSEVQVKETVTKFEEFLTSRGAEMVSKEDWGLKKMAYEIQNKKSGFYHLFEFK
     1        10        20        30        40       50        60
     :..**..*:...  .*::.*.::.:.::.*...**..*.....*.:.:.*.* :.:..*.*.:.**.

    66  70        80       90        100       110       120       130
Bbu MEGNNLKEFESRLKLIRNLLRYMILVKIVRKINTKKIKRRNFREFRDNIDKDSLKGASKVETPTGPE
Fjo VAGEVLIAFETEFRRDERVMRFLTVSLDKHAISWAERRRAKLKSTKA--------------------
    67 70        80       90        100       110
    :.*..*..**:..:....::*::.:......*:..:.:*...:..:.

    133   139
Bbu STDIQEK
Fjo -------
```

<u>bS18, Identity: 42/102 (41.2%), Similarity: 64/102 (62.7%)</u>

```
    1        10         20        30         40         50        60
Bbu MYKDRDTNQRDSRFENQQD-GFKKNSNFRFFK-RKSCKFCDSG-KHPDYKDFDFLKKFITEQGKILP
Fjo ----MSTIEQSAKGKKDGDIRYLTPLNIETNKTKKYCRFKKSGIKYIDYKDADFLLKFVNEQGKILP
        1         10        20        30        40        50        60
        ..*.::.::.:...* .:....*....* :*.*:*..** *:.****.***.**:.*******

    65   70        80        90    96
Bbu KRITGTSAKHQRRLALEIKRARYMALLPFVKK---
Fjo RRLTGTSLKYQRKVSVAVKRARHLALMPYVADLLK
    64   70        80        90      98
    :*:****.*:**::::.:****::**:*:*..
```

<u>bS21, Identity: 19/70 (27.1%), Similarity: 36/70 (51.4%)</u>

```
    1        10        20        30        40         50        60      68
Bbu MVTVTVDKNENLEKALKRFKRMIEKEAIIREWKRREYYEKPSTI-RVKKEKAFKRKQAKKVRKLKQKTNR
Fjo MLIIPIKDGENIDRALKRYKRKFDKTGTVRQLRARTAFIKPSVVKRAQIQKAAYIQTLKDSLES------
    1        10        20        30        40        50        60  64
    *:.:.:...**:::****:**..:*...:*:.:.*..:.***.: *.:.:**...:..*...:.
```

<u>16S RNA 3’-end</u>

```
Bbu UCGUAACAAGGUAGCCGUACUGGAAAGUGCGGCUGGAUCACCUCCUUU--
```

```
Fjo UCGUAACAAGGUAGCCGUACCGGAAGGUGCGGCUGGAACACCUCCUUUCU
    ******************** **** *********** **********
```

Core ASD sequence

Supplementary Note 3. Comparison of *Msm* bL37 to the *Bbu* uL30 N-terminal extension.

The *Msm* bL37 and *Bbu* uL30 N-terminal extension sequences align with a sequence identity of 21% :

```
Msm bL37    MAKRGRKKRDRKHSKANHGKRPNA
Bbu uL30    MIKRKLRLQLKKARFNASRSRSKN
            * **       *        *
```

The *Bbu* uL30 protein gene is in operon 171 with 24 other ribosomal proteins as well as other proteins. *Bbu* does not have a known bL37 protein sequence. The *Msm* uL30 protein gene is in operon 745 with 9 other ribosomal proteins and spans the nucleotides 1,565,535-1,565,720. The *Msm* bL37 protein gene is not in an operon with other genes and spans the nucleotides 1,998,031-1,998,105 of the *Msm* genome (Genbank ID: CP009494.1). It is not near the *Msm* uL30 protein gene and its neighboring protein genes in the genome are an anti-sigma factor (CDS: AIU07137.1) and an acetyl-CoA carboxylase (CDS: AIU07138.1).

Supplementary Note 4. Finding the bL38 ribosomal protein in *Bbu*.

A density in our *Bbu* 70S ribosome and 50S subunit cryo-EM maps was similar to the bL38 protein previously discovered in the Bacteroidetes[5] ribosomal large subunit, indicating that the bL38 protein is present in *Bbu*. There is no annotated bL38 protein sequence in the *Bbu* genome, so we first attempted to use the Bacteroidetes bL38 sequence from *Flavobacterium johnsoniae* (*Fjo*): MGSVIKKRRKRMSKKKHRKLLRRTRVQRRKLGK as a query sequence for a tblastn[6] search of the *Bbu* genome with a word size of 2 and no low complexity filter. This did not yield any plausible hits with a size near 50 aa residues upon translation of the genomic hits in 6 frames. We then performed mass spectrometric analysis of the proteins in our purified ribosomes. We identified all ribosomal proteins, including those missing in the cryo-EM densities, such as uS1 and uS2. We also identified the *Bbu* HPF protein and an uncharacterized protein in the 6 kDa range that was a promising candidate for bL38. An examination of the predicted Alphafold structure of this protein sequence showed a structure very similar to the C-α atom structure previously modeled manually into the cryo-EM density. The sidechains in the sequence matched the cryo-EM sidechain densities, thus confirming this to be the bL38 protein sequence.

A possible sequence alignment between *Bbu* and *Fjo* bL38 sequences shows only a 16.4% sequence identity, which explains why the *Fjo* query sequence tblastn search was unsuccessful:

```
Bbu    MAKISKNAQRVGSKELISRWGGKIIMKSKFENGKIKHYAECQTSKNTARKPRDLF

Fjo    MAKKTVASLQTSSKRLSKAIKMVKSPKTGAYIFVESIMAPELVDEFLKKK

       ***         ** *          *           *          *
```

The *Bbu* B31 bL38 protein is the previously uncharacterized gene *bb0162* (Uniprot ID: O51184).

Supplementary Table 1. The resolved 58 components of the *Bbu* 70S ribosome and 50S subunit.

Name	8FMW Chain ID	8FN2 (50S) Chain ID	Size (residues)	Modeled (residues)	Accession ID*	Comments
23S RNA	AA	A	2933	4-2932	AE000783.1	Nucleotides: 438267-435336
5S RNA	AB	B	112	1-112	AE000783.1	Nucleotides: 435312-435201
uL1	AC	C	226	6-226	O51353	Backbone model
uL2	AD	D	277	1-277	P94270	
uL3	AE	E	206	1-206	P94267	
uL4	AF	F	209	1-209	P94268	
uL5	AG	G	182	1-182	O51443	
uL6	AH	H	180	1-180	O51446	
bL9	AI	I	173	1-148	O51139	Partial backbone model (41-148)
uL10	AJ	J	162	1-162	O51352	
uL11	AK	K	143	5-143	O51354	
uL13	AL	L	146	2-146	O51314	
uL14	AM	M	122	1-122	O51441	
uL15	AN	N	145	1-145	O51450	
uL16	AO	O	138	1-138	O51438	
bL17	AP	P	123	1-121	O51456	
uL18	AQ	Q	119	1-119	O51447	
bL19	AR	R	121	1-117	O51642	
bL20	AS	S	115	2-115	O51206	
bL21	AT	T	103	1-103	O51719	
uL22	AU	U	120	2-116	P94272	
uL23	AV	V	98	1-98	P94269	
uL24	AW	W	101	1-101	O51442	
bL25	AX	X	182	2-182	O51727	
bL27	AY	Y	81	8-81	O51721	
bL28	AZ	Z	92	2-92	O51325	
uL29	Aa	a	65	1-65	O51439	
uL30	Ab	b	101	2-101	O51449	2 possible translation products
bL31	Ac	c	81	1-81	O51247	
bL32	Ad	d	60	2-60	O51646	Zn ion
bL33	Ae	e	59	9-59	O51357	
bL34	Af	f	51	1-50	P29220	
bL35	Ag	g	66	1-66	O51207	
bL36	Ah	h	37	1-37	O51452	Zn ion
bL38	Ai	i	Not known	1-46	Unidentified	Backbone model
E-tRNA	X	-			-	Mixture of tRNA populations
16S RNA	A	-	1538	1-1529	AE000783.1	Nucleotides: 444581-446118
uS3	C	-	293	6-226	P94273	67 C-terminal residues not seen
uS4	D	-	208	1-208	O51560	
uS5	E	-	165	8-165	O51448	
bS6	F	-	139	1-97	O51142	42 C-terminal residues not seen
uS7	G	-	157	1-157	O51347	
uS8	H	-	132	1-132	O51445	
uS9	I	-	136	6-136	O51313	
uS10	J	-	103	2-103	P94266	
uS11	K	-	130	14-130	O51454	13 N-terminal residues not seen
uS12	L	-	124	1-124	O51348	
uS13	M	-	125	1-114	O51453	11 C-terminal residues not seen

uS14	N	-	61	2-61	O51444	Zn ion
uS15	O	-	88	1-88	O51744	
bS16	P	-	86	1-83	O51638	
uS17	Q	-	84	3-84	O51440	
bS18	R	-	96	34-96	O51140	Zn ion, first 33 residues not seen
uS19	S	-	92	2-85	P94271	
bS20	T	-	85	1-85	P49394	
bS21	U	-	69	1-69	O51271	
bS22	V	-	31	2-28	AE000783.1	Nucleotides: 867605-867697
bbHPF	W	-	97	1-97	O51405	non-ribosomal protein

*Accession IDs are Genbank for RNA and Uniprot for proteins (except for the unannotated 30S ribosomal protein bS22). 30S ribosomal proteins uS1 and uS2 are present but uS1 is not resolved and uS2 is only partially resolved at very low threshold and therefore is not modeled.

Supplementary Table 2. Details of bacterial HPF structures with resolutions better than 3.5 Å that were used for sequence and structural comparisons.

PDB ID	Res. (Å)	Method	Year	Species (R, HPF)	Ref.	Component notes
4HEI	1.6	X-ray	2013	*-, Vch*	[10]	HPF alone, no ribosome
4Y4O	2.3	X-ray	2015	*Tth, Eco*	[11]	YfiA
6S0X	2.4	Cryo-EM	2019	*Sau, Sau*	[12]	HPF, erythromycin
7RQA	2.4	X-ray	2022	*Tth, Eco*	[13]	YfiA, tRNA analogs
7RQE	2.4	X-ray	2022	*Tth, Eco*	[13]	YfiA, tRNA analogs, chloramphenicol
3TQM	2.5	X-ray	2015	*-, Cbu*	[14]	HPF alone, no ribosome
7RQB	2.5	X-ray	2022	*Tth, Eco*	[13]	YfiA, tRNA analogs
7RQC	2.5	X-ray	2022	*Tth, Eco*	[13]	YfiA, tRNA analogs
7RQD	2.5	X-ray	2022	*Tth, Eco*	[13]	YfiA, tRNA analogs, chloramphenicol
2YWQ	2.6	X-ray	2008	*-, Tth*	[u]	HPF alone, no ribosome
6CFL	2.6	X-ray	2018	*Tth, Eco*	[15]	YfiA, D-lysyl-CAM
6XHX	2.6	X-ray	2021	*Tth, Eco*	[16]	YfiA, A2058 unmethylated, erythromycin
4V8I	2.7	X-ray	2011	*Tth, Eco*	[17]	YfiA
6CFK	2.7	X-ray	2018	*Tth, Eco*	[15]	YfiA, D-histidyl-CAM
5FDV	2.8	X-ray	2016	*Tth, Eco*	[18]	YfiA, Pyrrhocoricin
7MD7	2.8	X-ray	2021	*Tth, Eco*	[19]	YfiA, chloramphenicol
5FDU	2.9	X-ray	2016	*Tth, Eco*	[18]	YfiA, Metalnikowin I
5NGM	2.9	Cryo-EM	2017	*Sau, Sau*	[20]	HPF, 70S from 100S
6Y69	2.9	Cryo-EM	2020	*Eco, Eco*	[21]	HPF, TetracenomycinX
7M4Z	2.9	Cryo-EM	2021	*Aba, Aba*	[22]	HPF, eravacycline
6H4N	3.0	Cryo-EM	2018	*Eco, Eco*	[23]	HPF, 70S from 100S
4V8H	3.1	Cryo-EM	2012	*Tth, Eco*	[17]	HPF
6FKR	3.2	X-ray	2018	*Tth, Eco*	[24]	YfiA, Tur1A
5V8I	3.3	X-ray	2018	*Tth, Eco*	[np]	YfiA, no uS17
6GZQ	3.3	Cryo-EM	2018	*Tth, Tth*	[25]	HPF
5ZEP	3.4	Cryo-EM	2018	*Msm, Msm*	[4]	HPF
6DZI	3.5	Cryo-EM	2018	*Msm, Msm*	[26]	HPF (MPY)

Res. – Resolution, Ref. – Reference number, np – no associated publication, Species (R, HPF) refers to the species name abbreviation for ribosome (R) and HPF. Species name abbreviations are as follows: *Vch – Vibrio cholerae*, *Tth – Thermus thermophilus*, *Eco – Escherichia coli*, *Sau – Staphylococcus aureus*, *Cbu – Coxiella burnetii*, *Aba – Acinetabacter baumannii, Msm – Mycobacterium smegmatis*.

Supplementary Table 3. Structure-based pair-wise sequence alignment with bbHPF structure for known bacterial HPF structures.

ID	Aligned sequence
	β1 α1 β2 β3 β4 α2
	**bbb bbb** aaaaaaaaaaaaa **aaa bbbbbbb bbbbbbbb bbbbbbbb aaaaaaaaaaaaaaaaaaaaaaa**
Bbu	----MEPKIQ-TVNYSLNENEKNFILKKLEKFDTHIKKHIDNLKITIKKEH-----ELFKLDAHIHFN-W-GKIIHIREDGKILLNLIDSAIARLYKTATKEKEKKNNK----
Sau	----IRFEIH-GDNLTITDAIRNYIEEKIGK-LERYFNDVPNAVAHVKVKTYSN--SATKIEVTIPLK---NVTLRAEERNDDLYAGIDLINNKLERQVRKYKTRINRKSRDR
Tth	---MNIYKLI-GRNLEITDAIRDYVEKKLAR-LDRYQDGELMAKVVLSLAGSPHVEKKARAEIQVDLP---GGLVRVEEEDADLYAAIDRAVDRLETQVKRFRERRYVGKRHS
Eco1	-----TMNIT-SKQMEITPAIRQHVADRLAK-LEKWQTHLINPHIILSKEP-----QGFVADATINTP---NGVLVASGKHEDMYTAINELINKLERQLNKLQHKGEARRAA-
Eco2	----MQLNIT-GNNVEITEALREFVTAKFAK-LEQYFDRINQVYVVLKVEK-----VTHTSDATLHVN---GGEIHASAEGQDMYAAIDGLIDKLARQLTKHKDKLKQH----
Cbu	----MHIQMT-GQGVDISPALRELTEKKLHR-IQPCRDEISNIHIIFHINK-----LKKIVDANVKLP---GSTINAQAESDDMYKTVDLLMHKLETQLSKYKAK--------
Vch	----MQINIQ-GHHIDLTDSMQDYVHSKFDK-LERFFDHINHVQVILRVEK-----LRQIAEATLHVN---QAEIHAHADDENMYAAIDSLVDKLVRQLNKHKEKL-------
Msm	ERPHAEVVVK-GRNVEVPDHFRTYVSEKLSR-LERFDKTIYLFDVELDHERNRRQ-RKNCQHVEITARGR-GPVVRGEACADSFYTAFESAVQKLEGRLRRAKDRRKI-----
Aba	----MNIEIRTDKNIHNSERLITYVRAELTQEFQRHSERITHFSVHFSDENGDKG-GDKDIHCMIEARPSGLKPVAVHHKAGNIDASIHGAIEKLKRSLEHTFEKKE------
	: .. : . . : : : .. * :

Sequences aligned using structural alignment tool within UCSF Chimera. *Bbu – Borreliella burgdorferi* (O51405), *Sau - Staphylococcus aureus* (D2Z097), *Tth – Thermus thermophilus* (Q5SIS0), *Eco1 – Escherichia coli* YfiA (P0AD49), *Eco2 – Escherichia coli* HPF (P0AFX0), *Cbu – Coxiella burnetii* (Q83DI6), *Vch – Vibrio cholerae* (H9L4L9), *Msm – Mycobacterium smegmatis* (A0QTK6), *Aba – Acinetabacter baumannii* (V5V8V8). Uniprot IDs for proteins are in parentheses after species name. *Bbu* HPF (bbHPF) numbered secondary structure elements shown on top as α-helix (**a**) or β-strand (**b**). Bacterial HPF sequences are truncated within four residues of the bbHPF sequence. This table is distinct from Table 1 in the main manuscript in showing only residues that are resolved in the 3D structures of the HPFs in the alignment.

Supplementary Table 4. Predicted bS22 sequences in other *Borrelia* species.

Organism	Predicted bS22 protein sequence	Genome ID	Nucleotide Range
**Lyme borreliae**			
Borrelia burgdorferi	MPCGRKRKLKKISTHKRKKKRRKNRHKKKNK	AE000783.1	867605-867697
Borrelia afzelii	MPCGRKRKLKKISTHKRKKKRRKNRHKKKNK	CP042238.1	39086-39178
Borrelia garinii	MPCGRKRKLKKISTHKRKKKRRKNRHKKKNK	CP059009.1	871181-871273
Borrelia valaisiana	MPCGRKRKLKKISTHKRKKKRRKNRHKKKNK	CP009117.1	867482-867574
Borrelia mayonii	MPCGRKRKLKKISTHKRKKKRRKNRHKKKNK	CP015780.1	868560-868652
Borrelia maritima	MPCGRKRKLKKISTHKRKKKRRKNRHKKKNK	CP044535.1	865321-865413
Borrelia chilensis	MPCGRKRKLKKISTHKRKKKRRKNRHKKKNK	CP009910.1	864905-864997
Borrelia miyamotoi	MPCGRKRKLQKISTHKRKKKRRKNRHKKKNK	CP036914.1	39948-40040
**Relapsing fever borreliae**			
Borrelia duttonii	MPCGRKRKLKKISTHKRKKKRRKNRHKKKNK	CP000976.1	890138-890230
Borrelia recurrentis	MPCGRKRKLKKISTHKRKKKRRKNRHKKKNK	CP000993.1	891710-891802
Borrelia coriaceae	MPCGRKRKLKKISTHKRKKKRRKNRHKKKNK	CP075076.1	876376-876468
Borrelia crocidurae	MPCGRKRKLKKISTHKRKKKRRKNRHKKKNK	CP003426.1	876767-876859
Borrelia hermsii	MPCGRKRKLQKISTHKRKKKRRKNRHKKKNK	CP073148.1	879892-879984
Borrelia parkeri	MPCGRKRKLQKISTHKRKKKRRKNRHKKKNK	CP073159.1	878409-878501
Borrelia venezuelensis	MPCGRKRKLQKISTHKRKKKRRKNRHKKKNK	CP073220.1	878347-878439
Borrelia turicatae	MPCGRKRKLQKISTHKRKKKRRKNRHKKKNK	CP073192.1	877560-877652
**Reptile and echidna *Borrelia***			
Candidatus Borrelia tachyglossi	MPCGRKRKLKKISTHKRKKKRRKNRHKKKNK	CP025785.1	894477-894569
	********* *********************		

All sequences identified through a tblastn[6] search using the *Bbu* bS22 sequence.

Supplementary Table 5. Bacterial 50S assembly intermediate structures compared to the *Bbu* 50S subunit structure.

EMDB ID	Resolution (Å)	Threshold used	Organism	Description
8274	5.8	0.014	*Bsu*	44.5S YsxC class I[27]
8275	6.2	0.014	*Bsu*	44.5S YsxC class II[27]
8276	6.5	0.014	*Bsu*	45S YphC[27]
8440	4.5	0.030	*Eco*	Class B[28]
8441	3.7	0.030	*Eco*	Class C[28]
8442	4.5	0.030	*Eco*	Class C1[28]
8443	4.6	0.030	*Eco*	Class C2[28]
8444	4.0	0.030	*Eco*	Class C3[28]
8445	4.0	0.030	*Eco*	Class D[28]
8446	4.7	0.030	*Eco*	Class D1[28]
8447	4.9	0.030	*Eco*	Class D2[28]
8448	4.6	0.030	*Eco*	Class D3[28]
8449	4.7	0.030	*Eco*	Class D4[28]
8450	3.7	0.030	*Eco*	Class E[28]
8451	4.3	0.030	*Eco*	Class E1[28]
8452	4.5	0.030	*Eco*	Class E2[28]
8453	5.0	0.030	*Eco*	Class E3[28]
8455	4.5	0.030	*Eco*	Class E4[28]
8456	4.9	0.030	*Eco*	Class E5[28]

Bsu – Bacillus subtilis, *Eco – Escherichia coli*.

Supplementary Table 6. Structures of bacterial ribosomes with antibiotics that were used to help generate *Bbu* 70S ribosome antibiotic bound models by structural analogy.

PDB ID	Res. (Å)	Method	Year	Species	Ref.	Antibiotic, other notes
5J5B	2.8	X-ray	2016	*Eco*	[29]	Tetracycline,
5J7L	3.0	X-ray	2016	*Eco*	[29]	Tetracycline, at 2 sites, U1052 mutation
4V9A	3.3	X-ray	2013	*Tth*	[30]	Tetracycline
1HNW	3.4	X-ray	2000	*Tth*	[31]	Tetracycline
1I97	3.5	X-ray	2001	*Tth*	[32]	Tetracycline
6S0Z	2.3	Cryo-EM	2019	*Sau*	[12]	Erythromycin, ΔR88,ΔA89 uL22
6S0X	2.4	Cryo-EM	2029	*Sau*	[12]	Erythromycin, ΔR88,ΔA89 uL22
6XHX	2.6	X-ray	2021	*Tth*	[16]	Erythromycin, A2058 unmethylated, YfiA
1YI2	2.7	X-ray	2005	*Hma*	[33]	Erythromycin, G2099A
6ND6	2.9	X-ray	2019	*Tth*	[34]	Erythromycin, A-, P-, E-tRNAs
7B5K	2.9	Cryo-EM	2021	*Eco*	[35]	Erythromycin, P-site tRNA nascent chain
7NSO	2.9	Cryo-EM	2021	*Eco*	[36]	Erythromycin, ErmDL
7Q4K	3.0	Cryo-EM	2022	*Eco*	[u]	Erythromycin, streptococcal MsrDL
4V7X	3.0	X-ray	2010	*Tth*	[37]	Erythromycin
4V7U	3.1	X-ray	2010	*Eco*	[37]	Erythromycin
4WFN	3.5	X-ray	2017	*Dra*	[38]	Erythromycin, uL22 3-residue insertion
1JZY	3.5	X-ray	2001	*Dra*	[39]	Erythromycin
7NSP	3.5	Cryo-EM	2021	*Eco*	[36]	Erythromycin, ErmDL, A-, P-tRNA
5JTE	3.6	Cryo-EM	2016	*Eco*	[40]	Erythromycin, ErmBL, A-, P-, E-tRNA
5JU8	3.6	Cryo-EM	2016	*Eco*	[40]	Erythromycin, ErmBL, P-, E-tRNA
3J7Z	3.9	Cryo-EM	2014	*Eco*	[41]	Erythromycin, ErmCL
3J5L	6.6	Cryo-EM	2014	*Eco*	[42]	Erythromycin, ErmBL
5DOY	2.6	X-ray	2015	*Tth*	[43]	Hygromycin A, A-, P-, E-tRNA
5DM7	3.0	X-ray	2015	*Dra*	[43]	Hygromycin A
5DOX	3.1	X-ray	2015	*Tth*	[44]	Hygromycin A

Res. – Resolution, Ref. – Reference number, u - Unpublished structure, Species refers to the species name abbreviation for ribosome that are as follows: *Eco – Escherichia coli*, *Tth – Thermus thermophilus*, *Sau – Staphylococcus aureus*, *Hma – Haloarcula marismortui*, *Dra – Deinococcus radiodurans.*

Supplementary Table 7. Long-gradient LC-MS/MS analysis of proteins in the *Bbu* 70S ribosome.

Protein name	Percent coverage	Pip (%)	Total spectrum count	Protein Name	Percent coverage	Pip (%)	Total spectrum count
bS1	12	100	7	uL1	82	100	135
uS2	90	100	128	uL2	85	100	175
uS3	87	100	227	uL3	79	100	105
uS4	90	100	148	uL4	68	100	30
uS5	85	100	94	uL5	76	100	98
bS6	83	100	106	uL6	71	100	82
uS7	88	100	97	bL7/bL12	68	100	66
uS8	97	100	135	bL9	79	100	103
uS9	81	100	92	uL10	90	100	54
uS10	100	100	65	uL11	94	100	82
uS11	77	100	54	uL13	90	100	41
uS12	74	100	56	uL14	99	100	81
uS13	79	100	101	uL15	85	100	96
uS14	56	100	15	uL16	80	100	141
uS15	76	100	104	bL17	83	100	46
bS16	85	100	50	uL18	74	100	50
uS17	86	100	44	bL19	74	100	46
bS18	84	100	74	bL20	68	100	15
uS19	86	100	34	bL21	75	100	12
bS20	84	100	41	uL22	84	100	30
bS21	43	100	32	uL23	52	100	10
bS22	-	-	-	uL24	75	100	61
HPF	67	100	29	bL25	93	100	117
				bL27	56	100	52
				bL28	89	100	86
				uL29	92	100	39
				uL30	75	100	41
				bL31	91	100	56
				bL32	53	100	39
				bL33	61	100	34
				bL34	47	100	10
				bL35	52	100	23
				bL36	54	100	11
				bL38	56	100	15

Pip – Protein identification probability determined by Scaffold5.

Supplementary Table 8. Alphafold predicted Local Distance Difference Test (pLDDT) score statistics for starting models for proteins included in the reported structures.

Protein name	Average	Max.	Min.	Protein Name	Average	Max.	Min.
uS3	82.13	97.12	39.72	uL1	85.25	93.79	44.93
uS4	91.62	97.67	34.89	uL2	91.69	97.31	56.54
uS5	92.28	98.02	36.55	uL3	90.84	97.79	61.36
bS6	83.38	97.89	39.53	uL4	92.00	97.77	74.59
uS7	92.13	97.58	49.85	uL5	89.74	97.34	48.42
uS8	93.28	98.20	40.16	uL6	89.91	96.25	50.30
uS9	91.08	97.69	43.80	bL9	85.60	97.06	40.29
uS10	88.07	96.56	39.07	uL10	74.00	85.29	43.38
uS11	89.27	97.14	50.76	uL11	78.05	88.51	37.14
uS12	94.26	98.22	79.90	uL13	88.83	97.43	39.48
uS13	89.43	97.21	66.20	uL14	96.49	98.72	87.62
uS14	91.59	95.23	74.95	uL15	88.24	96.70	50.40
uS15	92.74	97.94	66.48	uL16	93.52	98.03	52.53
bS16	88.75	97.21	40.51	bL17	92.57	98.35	41.20
uS17	90.47	96.75	45.62	uL18	90.38	95.03	62.67
bS18	79.41	96.67	42.18	bL19	84.67	97.33	34.56
uS19	87.53	96.85	46.03	bL20	91.77	97.19	59.31
bS20	93.33	97.68	56.78	bL21	91.13	96.63	63.77
bS21	90.36	97.13	63.82	uL22	89.99	97.13	32.21
bS22	89.38	55.01	97.69	uL23	92.97	97.95	77.31
HPF	89.82	95.81	53.19	uL24	90.30	95.37	73.41
				bL25	92.57	97.18	37.76
				bL27	91.97	98.40	60.98
				bL28	89.60	94.99	59.27
				uL29	83.18	93.34	53.46
				uL30	95.24	98.18	70.65
				bL31	62.42	79.34	49.20
				bL32	88.74	97.46	54.50
				bL33	79.94	92.45	44.28
				bL34	92.63	98.35	73.06
				bL35	91.53	95.86	66.98
				bL36	92.38	96.52	59.86
				bL38	74.16	88.94	42.44

Max. – maximum pLDDT score of any residue in protein, Min. – minimum pLDDT score of any residue in protein. pLDDT scores range between 0 – 100, with 100 indicating the highest confidence.

Supplementary Table 9. Details of RNA structure overlays for antibiotic-bound structures reported.

PDB ID	Chain aligned	Pruned equivalent atom pairs	RMSD (Å)	Antibiotic bound
6s0z	A (23S RNA)	1964	1.032	ERY
6s0x	A (23S RNA)	1252	1.034	ERY
6xhx	1A (23S RNA)	2063	1.035	ERY
6xhx	2A (23S RNA)	2094	1.035	ERY
1yi2	0 (23S RNA)	1416	1.170	ERY
6nd6	1A (23S RNA)	2075	1.064	ERY
6nd6	2A (23S RNA)	2092	1.062	ERY
7b5k	A (23S RNA)	2127	0.974	ERY
7nso	A (23S RNA)	2226	0.856	ERY
7q4k	BA (23S RNA)	2177	0.934	ERY
4v7x	BA (23S RNA)	2081	1.027	ERY
4v7x	DA (23S RNA)	2078	1.024	ERY
4v7u	BA (23S RNA)	2146	0.923	ERY
4v7u	DA (23S RNA)	2109	0.984	ERY
4wfn	X (23S RNA)	2077	0.967	ERY
1jzy	A (23S RNA)	1963	1.109	ERY
7nsp	A (23S RNA)	2194	0.874	ERY
5jte	BA (23S RNA)	2161	0.894	ERY
5ju8	BA (23S RNA)	2158	0.898	ERY
3j7z	A (23S RNA)	2132	0.984	ERY
3j5l	A (23S RNA)	2145	0.924	ERY
5dm7	X (23S RNA)	2092	0.992	HGR
5dox	1A (23S RNA)	2054	1.058	HGR
5dox	2A (23S RNA)	2067	1.053	HGR
5doy	1A (23S RNA)	2017	1.155	HGR
5doy	2A (23S RNA)	2023	1.139	HGR
5j5b	AA (16S RNA)	607	1.124	TAC
5j5b	BA (16S RNA)	904	1.022	TAC
5j7l	AA (16S RNA)	600	1.091	TAC
5j7l	BA (16S RNA)	907	0.985	TAC
4v9a	AA (16S RNA)	967	1.112	TAC
4v9a	CA (16S RNA)	1101	1.167	TAC
1hnw	A (16S RNA)	815	1.078	TAC
1i97	A (16S RNA)	751	1.207	TAC

All overlays done using Matchmaker in ChimeraX[45], ERY – erythromycin, HGR – hygromycin A, TAC – Tetracycline.

Supplementary Table 10. Details of RNA structure overlays for HPF-bound structures.

PDB ID	Chain aligned (16S RNA)	Pruned equivalent atom pairs	RMSD (Å)
4Y4O	1a	931	1.147
4Y4O	2a	1135	1.159
6S0X	a	478	1.329
7RQA	1a	934	1.153
7RQA	2a	1136	1.172
7RQE	1a	973	1.092
7RQE	2a	1159	1.149
7RQB	1a	958	1.115
7RQB	2a	1145	1.153
7RQC	1a	953	1.123
7RQC	2a	1146	1.162
7RQD	1a	972	1.115
7RQD	2a	1161	1.155
6CFL	1a	962	1.089
6CFL	2a	1167	1.129
6XHX	1a	950	1.115
6XHX	2a	1144	1.160
4V8I	AA	972	1.121
4V8I	CA	1118	1.131
6CFK	1a	958	1.096
6CFK	2a	1163	1.135
5FDV	1a	967	1.162
5FDV	2a	1134	1.157
7MD7	1a	955	1.097
7MD7	2a	1164	1.156
5FDU	1a	952	1.184
5FDU	2a	1125	1.180
5NGM	Aa	802	1.096
6Y69	a	1237	0.983
7M4Z	a	933	1.256
6ERI	BA	1199	1.011
6H4N	a	1249	0.967
4V8H	AA	831	1.089
4V8H	CA	894	1.069
6FKR	1a	973	1.179
6FKR	2a	1144	1.157
5V8I	1a	1014	1.080
5V8I	2a	1158	1.128
6GZQ	A2	833	1.272
5MMM	a	1195	0.995
5X8P	a	1174	1.047
5ZEP	a	1131	1.032
6DZI	h	1110	1.171

All overlays done using Matchmaker in ChimeraX[45].

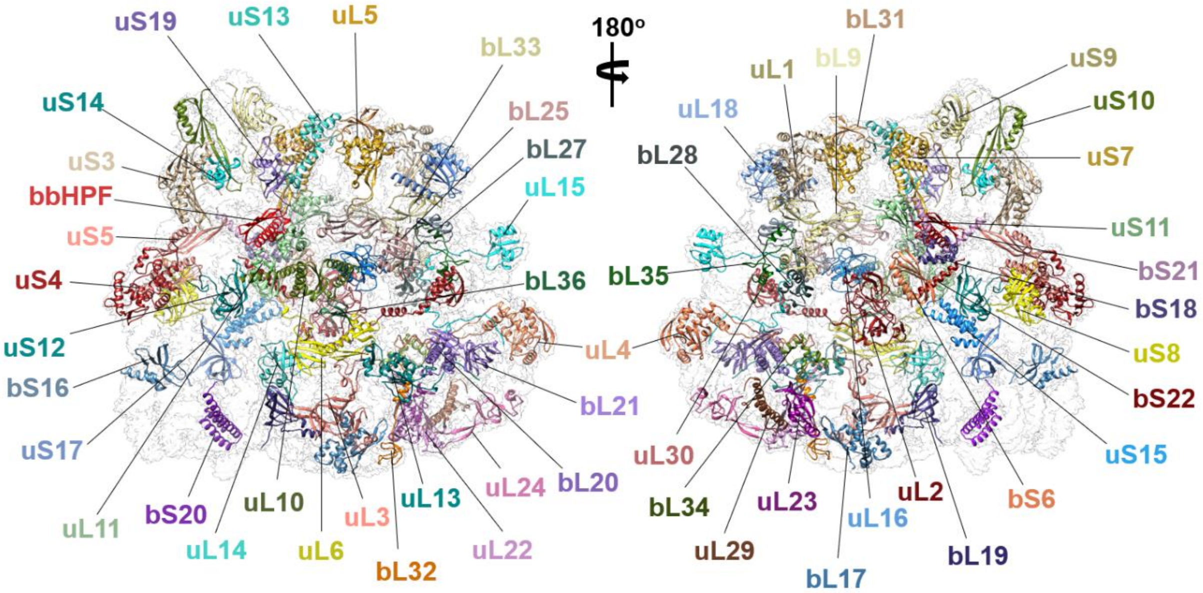

Supplementary Figure 1. Protein components of the hibernating *Bbu* 70S ribosome and their labeled models fit in the cryo-EM density.

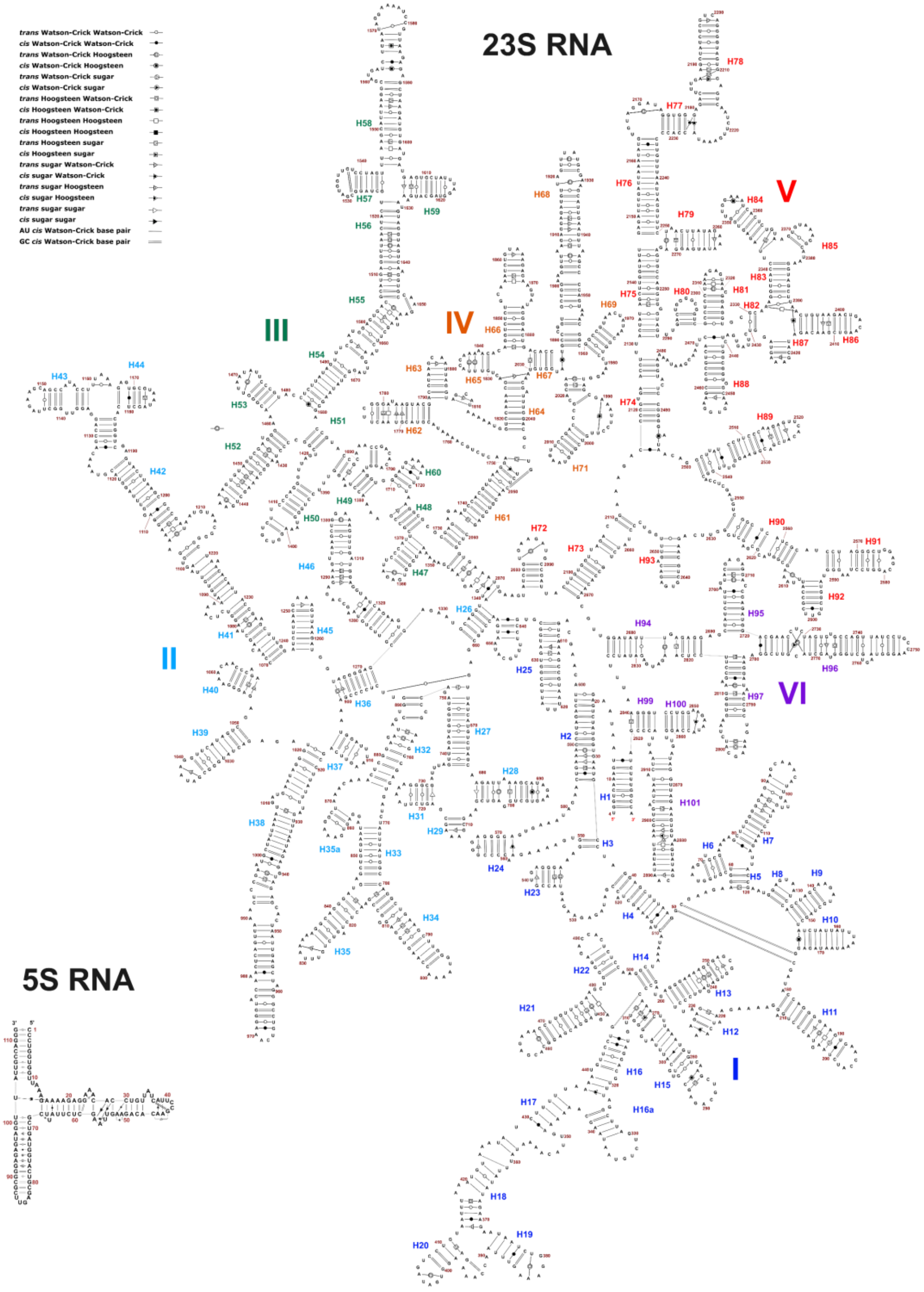

Supplementary Figure 2. 23S RNA and 5S RNA secondary structure in the *Bbu* 70S ribosome.

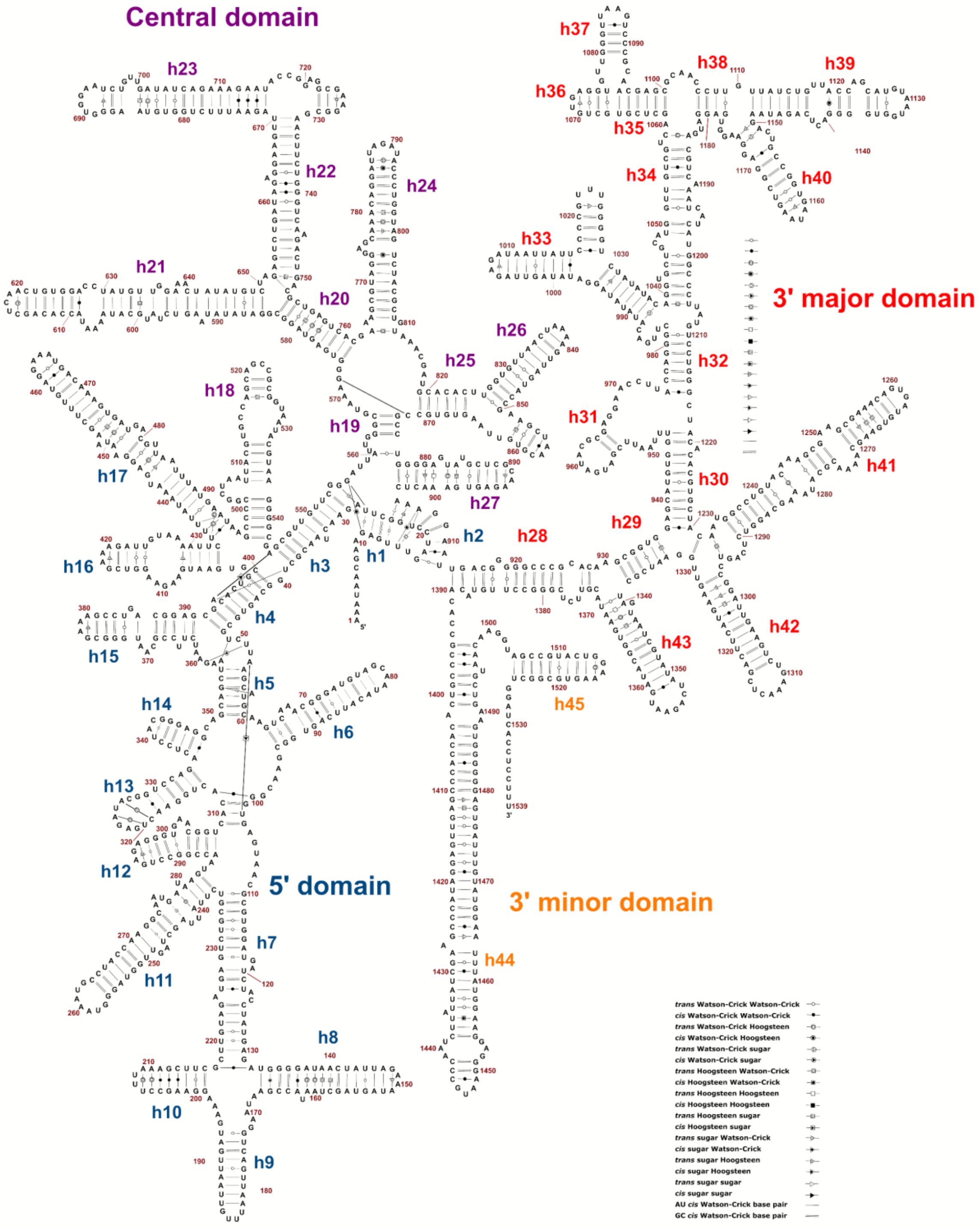

Supplementary Figure 3. 16S RNA secondary structure in the *Bbu* 70S ribosome.

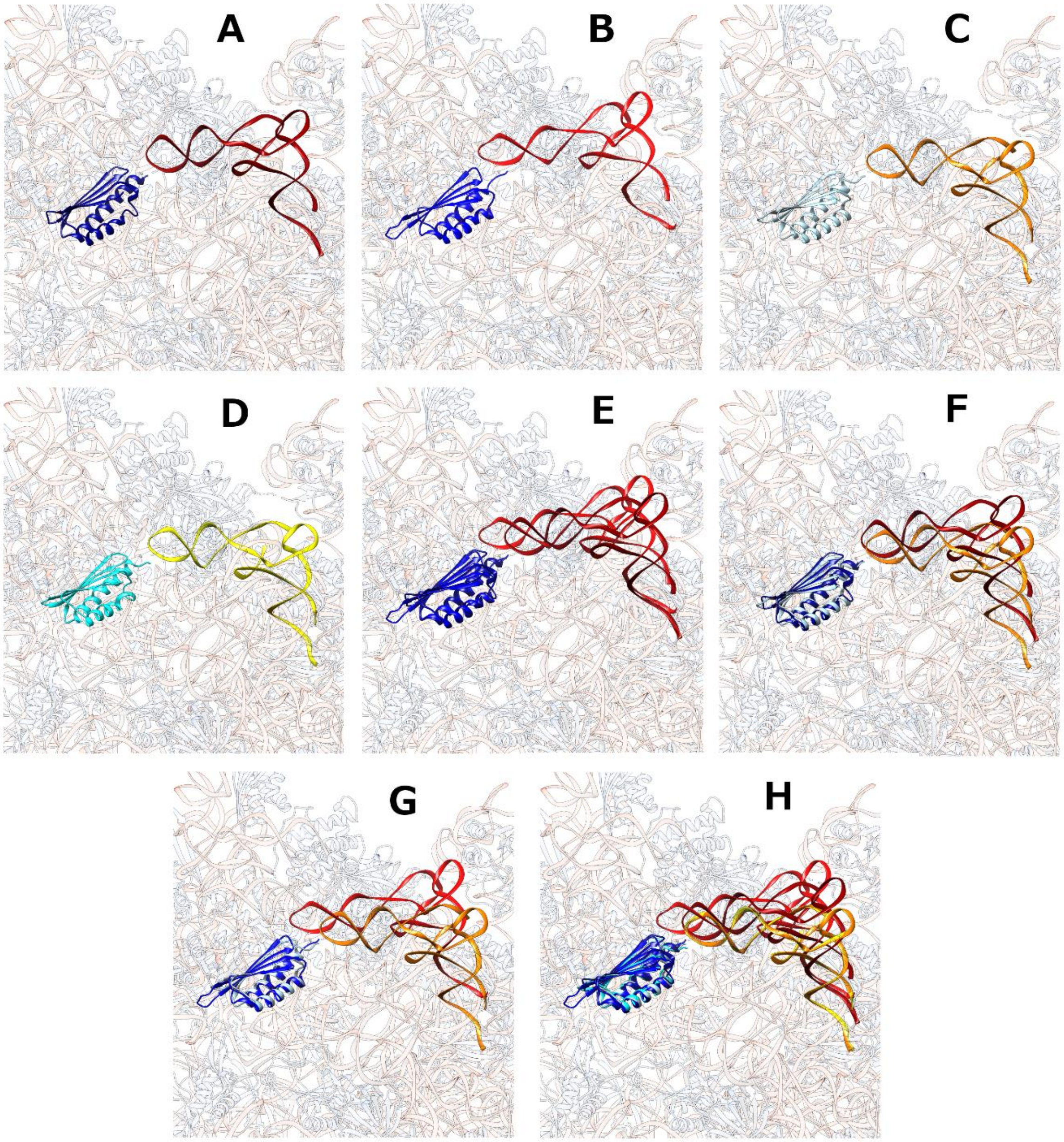

Supplementary Figure 4. Variability in relative orientation of HPF protein and E-tRNA bound to 70S ribosomes. (A) *Bbu* (this study); **(B)** *Msm* (PDB ID: 5ZEP); **(C)** *Eco* (PDB ID: 6H4N); **(D)** *Eco* (PDB ID: 6Y69); **(E)** Overlay of (A) and (B); **(F)** Overlay of (A) and (C); **(G)** Overlay of (B) and (C); **(H)** Overlay of (A)-(D). Background transparent structure is the *Bbu* 70S ribosome.

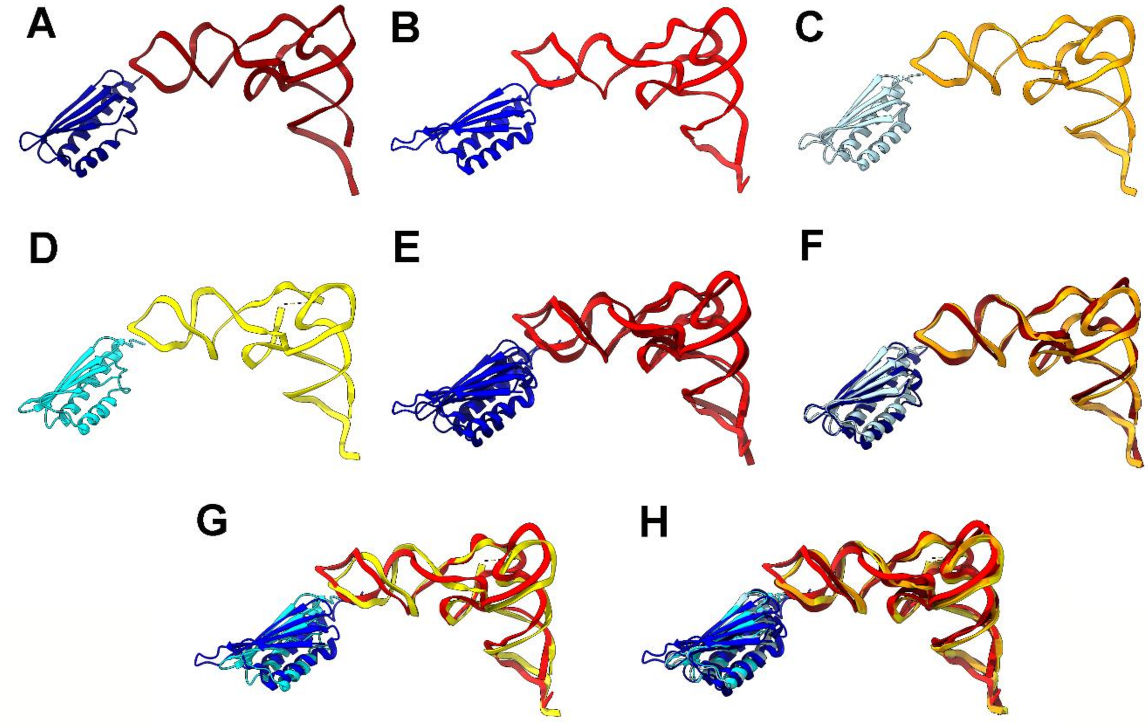

Supplementary Figure 5. Adjustment in positioning for both HPF and E-tRNA within their binding sites reduces their overall variability with respect to the 70S ribosome. (A) *Bbu* (this study); **(B)** *Msm* (PDB ID: 5ZEP); **(C)** *Eco* (PDB ID: 6H4N); **(D)** *Eco* (PDB ID: 6Y69); **(E)** Overlay of (A) and (B); **(F)** Overlay of (A) and (C); **(G)** Overlay of (B) and (C); **(H)** Overlay of (A)-(D). All structures overlaid using the 16S ribosomal RNA.

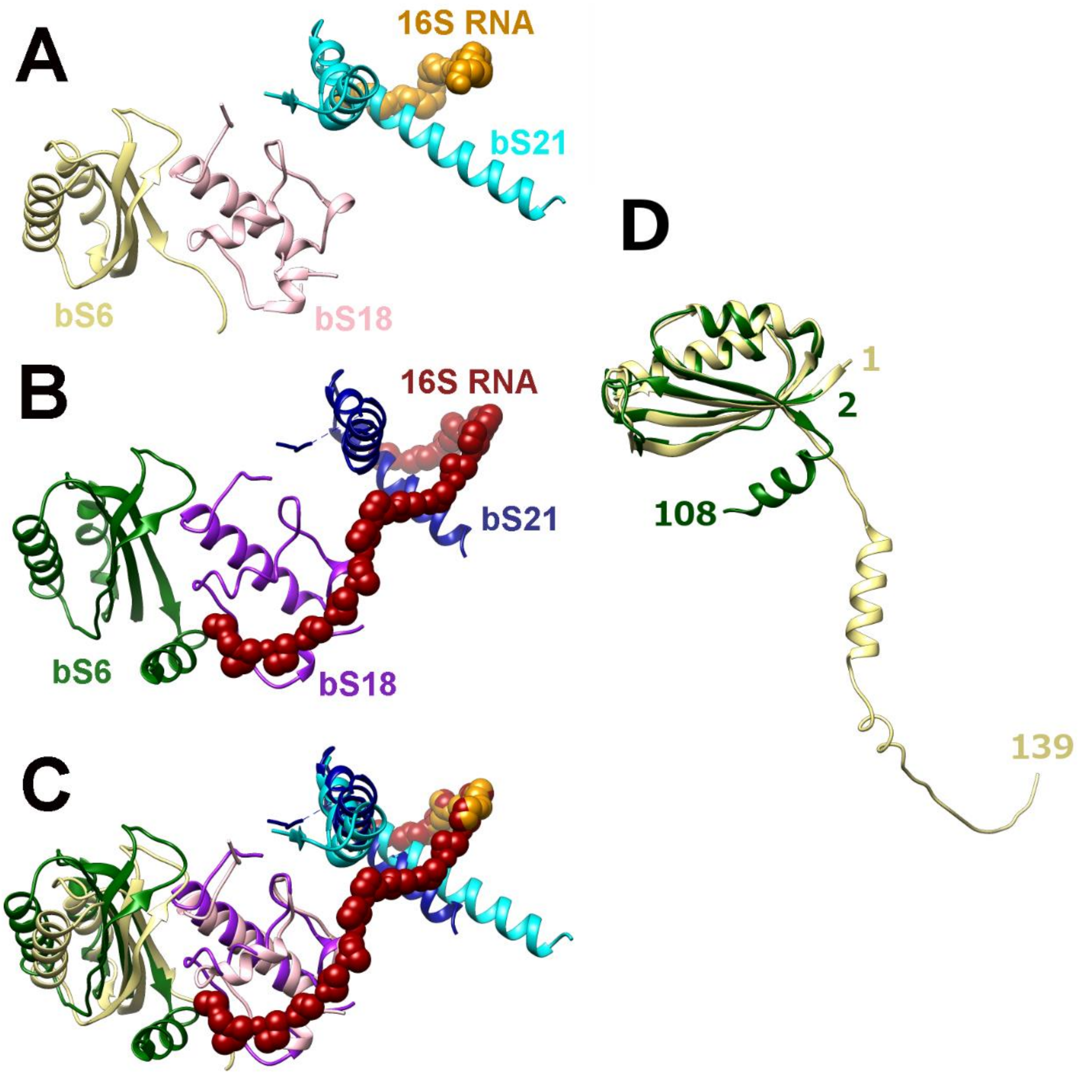

Supplementary Figure 6. A binding pocket formed by bS6, bS18 and bS21 implicated in Anti-Shine Dalgarno (ASD) sequence sequestration. (A) The binding pocket in *Bbu* (this study). **(B)** The binding pocket in *Fjo* with the sequestered ASD (PDB ID: 7JIL[5])**. (C)** Overlay of the *Bbu* and *Fjo* binding pockets. **(D)** Overlay of experimentally determined *Fjo* bS6 structure (green) and full-length *Bbu* bS6 Alphafold-predicted structure (yellow) showing likely presence of ASD interacting helix in *Bbu*. For *Bbu*, the 16S RNA minimal backbone -[O5'-C5'-C4'-C3'-O3'-P]- is shown in orange spheres, bS6 is in yellow, bs18 is in pink, and bS21 is in cyan. For *Fjo*, the 16S RNA minimal backbone is shown in red spheres, bS6 is in green, bs18 is in purple, and bS21 is in blue.

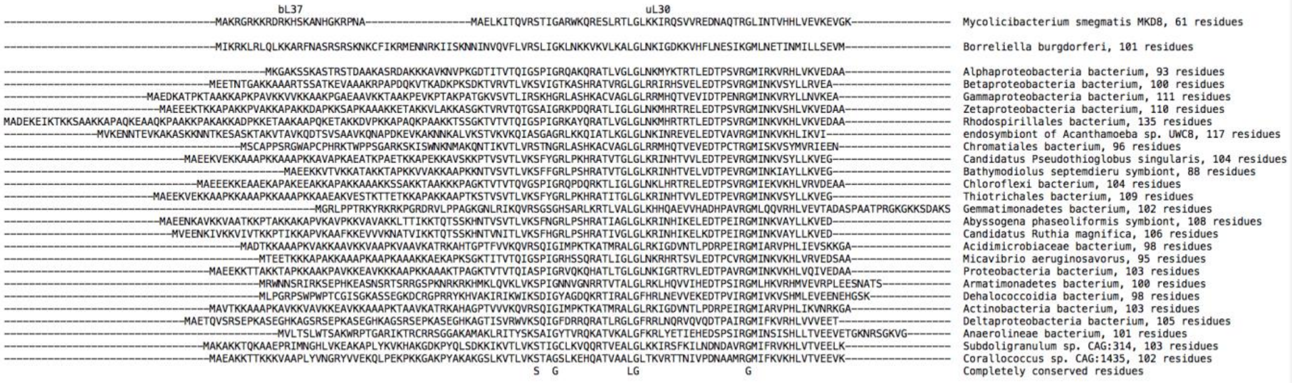

Supplementary Figure 7. Long uL30 proteins identified in other bacterial species. *Msm* ul30 and bL37 sequences are shown on top and the *Bbu* uL30 sequence is shown second for reference.

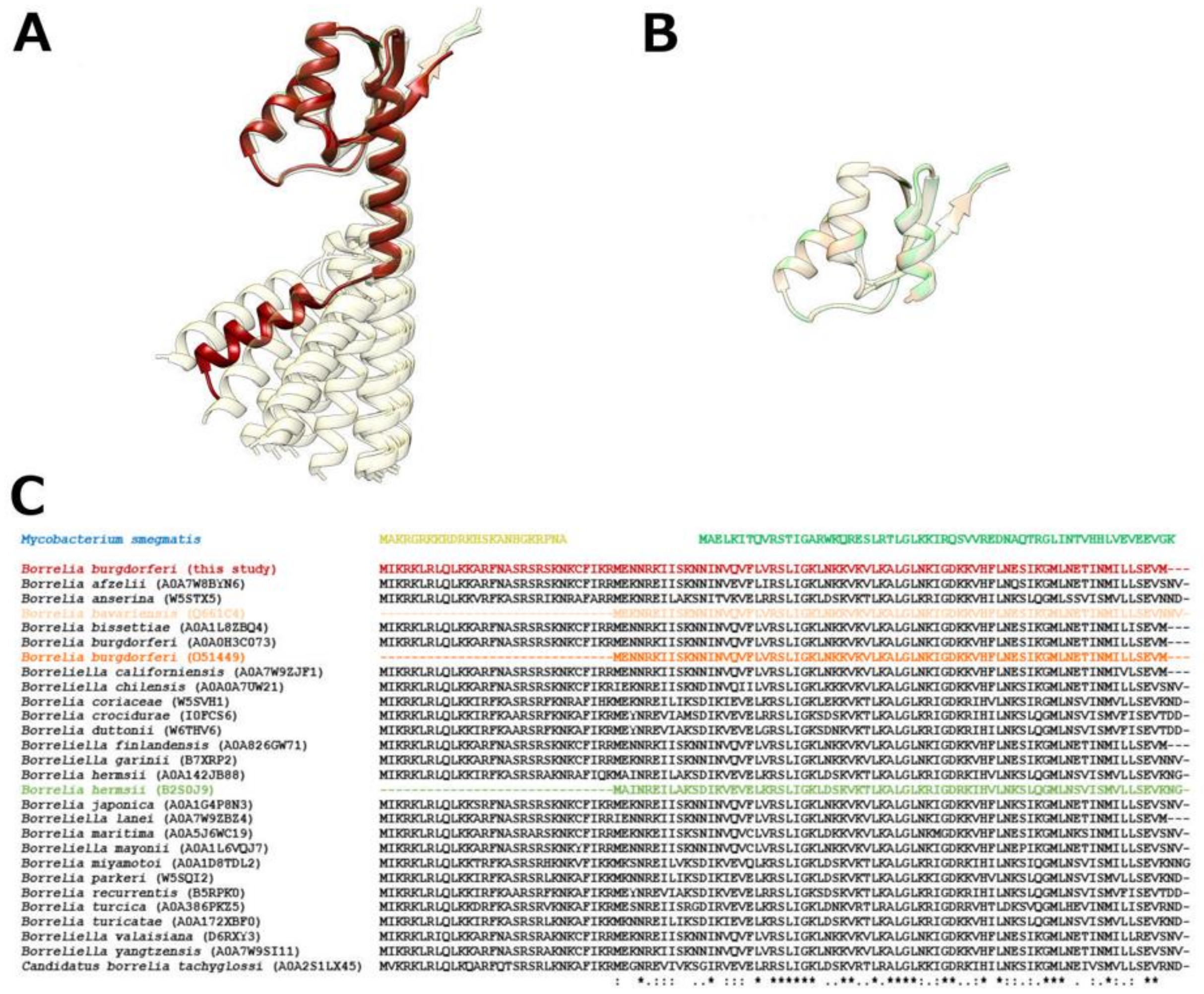

Supplementary Figure 8. Two types of longer uL30 proteins in *Borreliella* species. (A) Alphafold structure predictions for longer *Borreliella* species uL30 proteins, *Bbu* uL30 is shown in dark red; **(B)** Alphafold structure predictions for three shorter *Borreliella* species uL30 proteins; **(C)** Sequence alignment of *Borreliella* species uL30 proteins shown in A and B with *Msm* uL30 and bL37 also aligned for reference. Colors are consistent between models shown in **A** and **B** and sequences shown in **C** except the longer uL30 models shown in khaki color in **A** are shown in black text for their sequence in **C**.

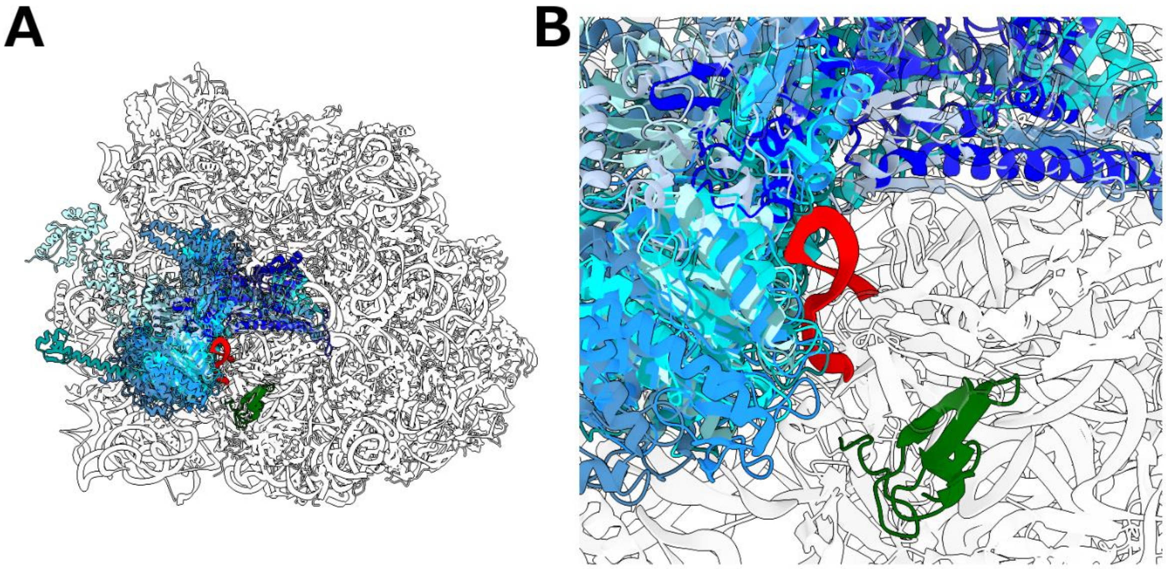

Supplementary Figure 9. The proximity of *Bbu* bL38 (dark green) to representative GTPase domain protein interactions with the sarcin-ricin loop (SRL, red). (A) View with full ribosome shown in transparent grey; **(B)** Zoomed in view for region near bL38 and the SRL. The GTPase domain proteins shown are tetracycline resistance protein TetM (PDB ID: 3J9Y[46]) in cyan, translation initiation factor IF-2 (PDB ID: 3JCJ[47]) in dark turquoise, elongation factor Tu 2 (PDB ID: 5AFI[48]) in cadet blue, elongation factor 4 (PDB ID: 5J8B[49]) in dark cyan, selenocysteine-specific elongation factor (PDB ID: 5LZD[50]) in pale turquoise, GTPase ObgE/CgtA (PDB ID: 7BL4[51]) in light steel blue, peptide chain release factor 1 and peptide chain release factor 3 (PDB ID: 7M5D) in steel blue, elongation factor G (PDB ID: 7N2V[52]) in dodger blue, and GTPase Hflx (PDB ID: 7YLA) in blue. All color descriptors are from ChimeraX[45] and all overlays are done using the Matchmaker module in ChimeraX[45] for the 23S RNA.

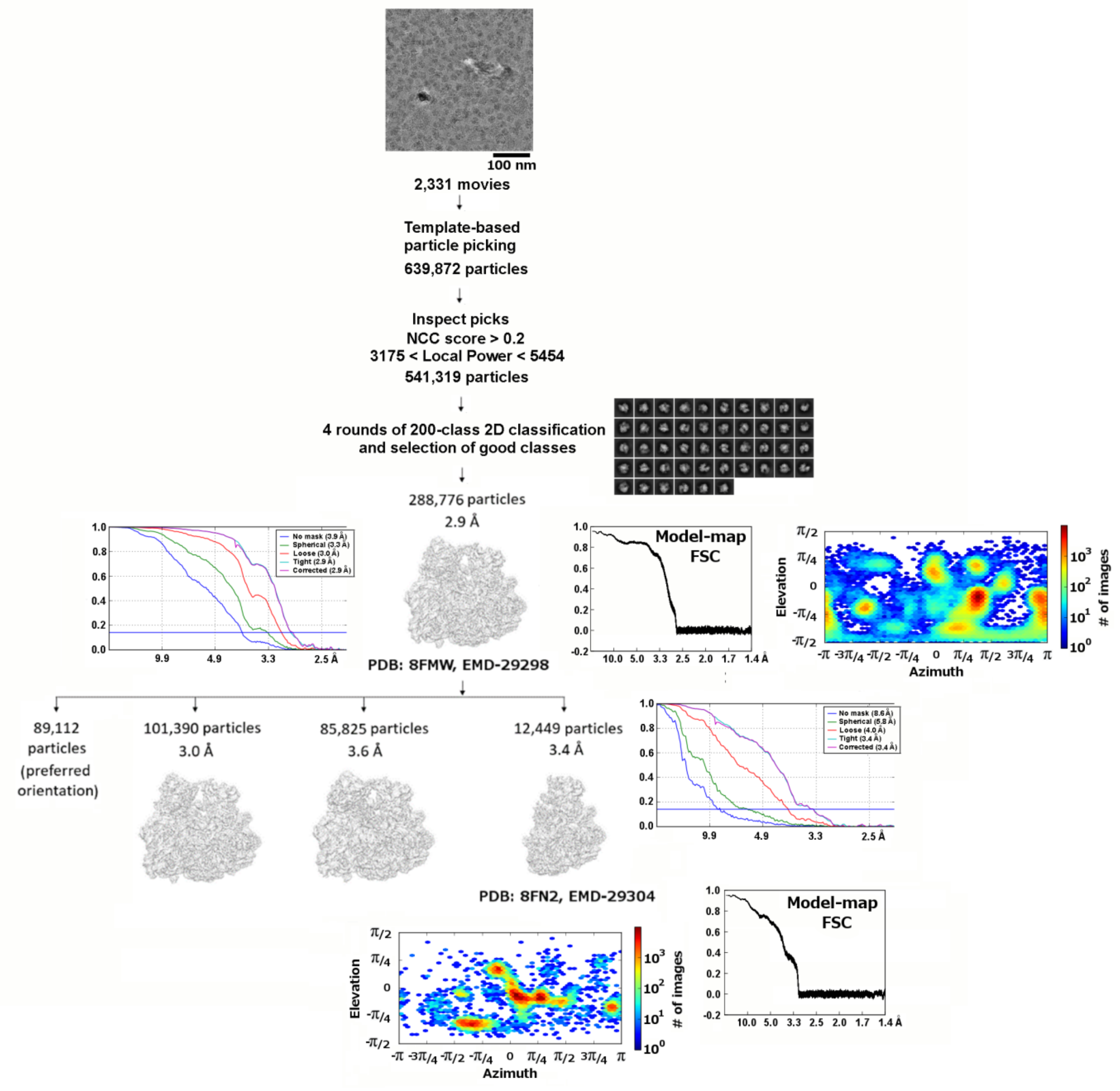

Supplementary Figure 10. The image processing and 3D classification flowchart of cryo-EM particles for the *Bbu* ribosome. From the final set of 288,776 particles, the classes with 101,390 particles and 85,825 particles did not show any appreciable difference with each other or for subclasses generated from them. The PDB IDs and EMD IDs for the two volumes and corresponding models deposited are shown. All densities are shown at a common threshold value of 0.18. The model-map FSC plot (black dots), the FSC plots with varied masking (colored lines), and the distribution of image projection orientations are shown in proximity to the respective deposited maps. Source data are provided as a Source Data file.

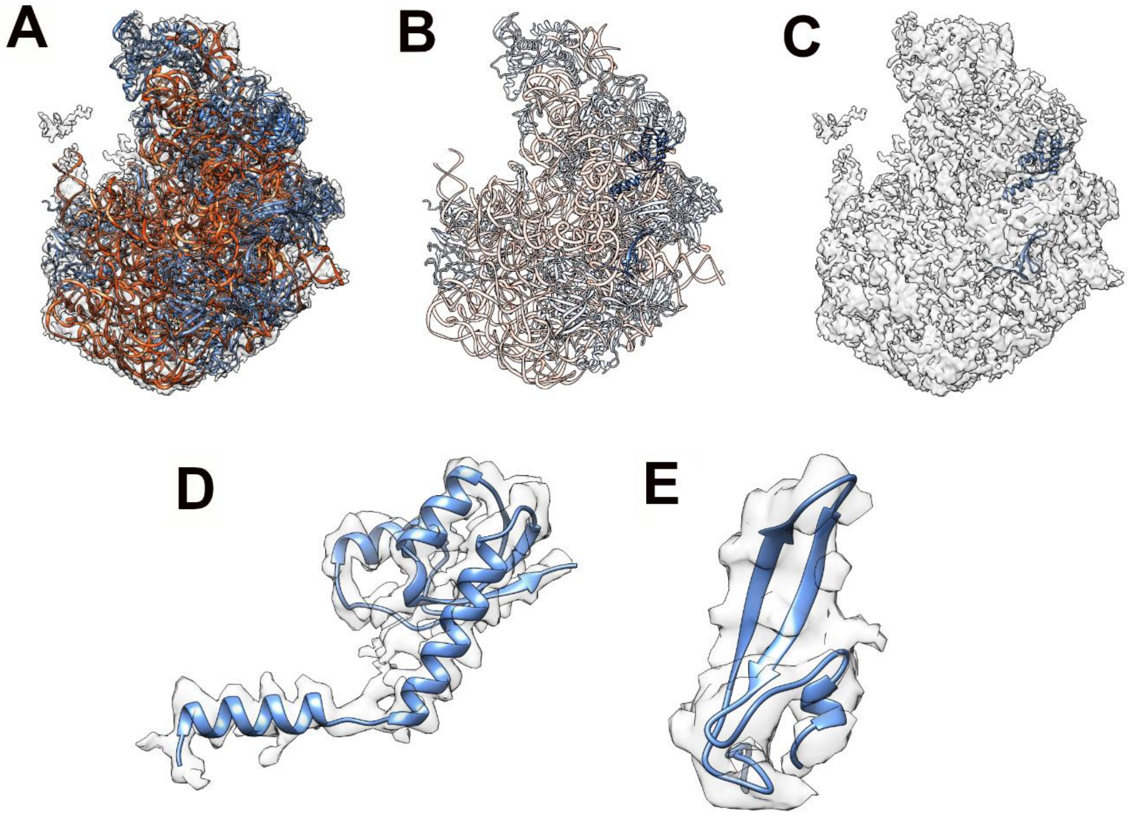

Supplementary Figure 11. Large subunit 50S structure for *Bbu* showing that its distinct ribosomal protein components are present in the isolated large subunit and are not specific to the 70S assembly. (A) The *Bbu* 50S density at 3.4 Å resolution in transparent khaki with its fitted model in opaque orange-red for RNA and in opaque blue for proteins; **(B)** The *Bbu* 50S model in transparent depiction except for uL30 and bL38 proteins shown in opaque blue; **(C)** The *Bbu* 50S density in transparent khaki with uL30 and bL38 proteins shown in opaque blue; **(D)** The model and excised 50S density for uL30; **(E)** The model and excised 50S density for bL38.

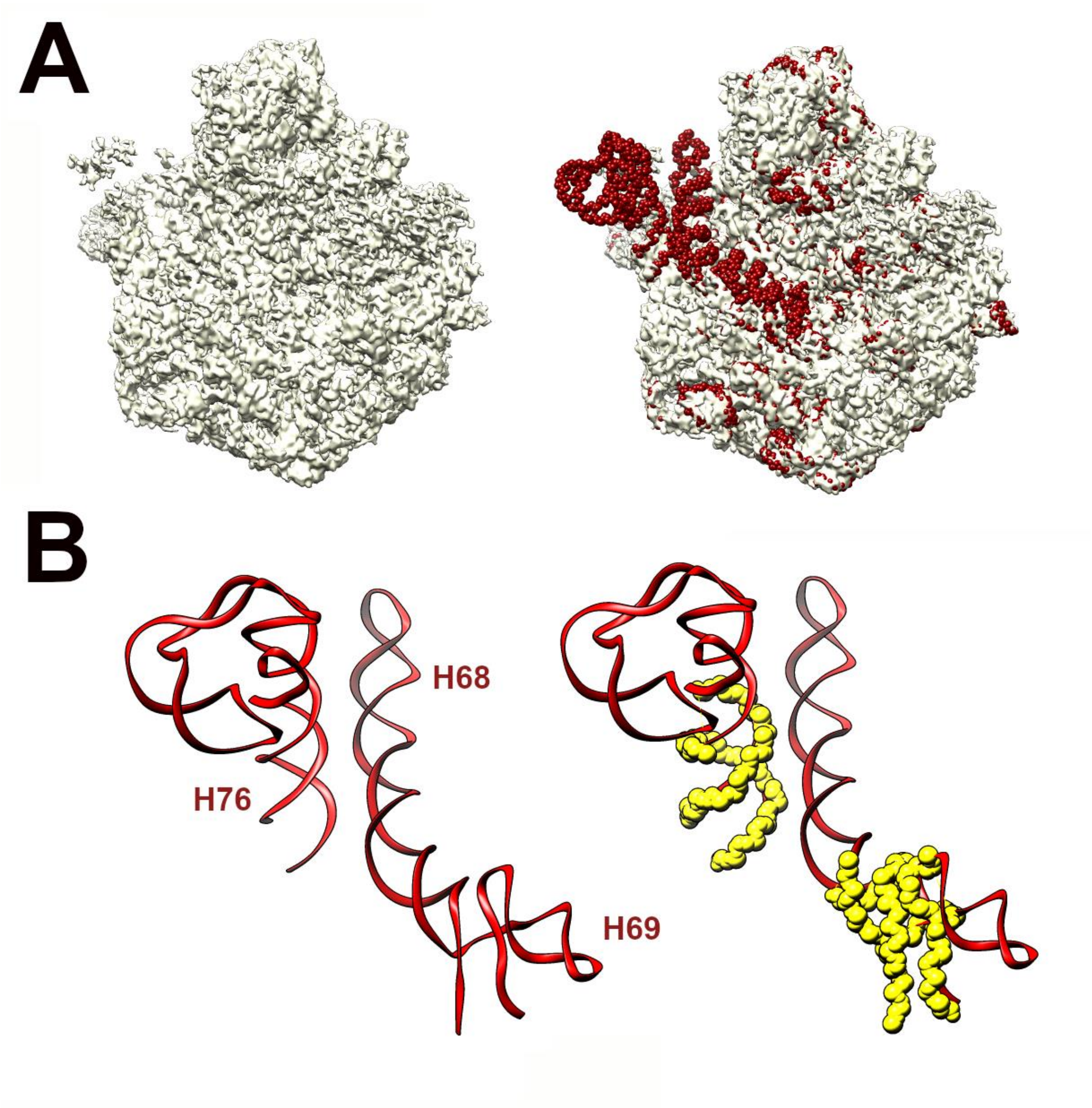

Supplementary Figure 12. Disordering of 23S rRNA helices H68 and H69 in the *Bbu* large subunit 50S density. (A) The *Bbu* 50S density at 3.4 Å resolution in transparent khaki shown at a threshold of 0.15 alone (left) and with the fitted 70S structure 23S rRNA model with backbone atoms shown in red spheres (right) indicating disorder in specific 23S rRNA regions; **(B)** The specific 70S model *Bbu* 23S rRNA regions shown in red ribbons (left) and with the modeled backbone atoms in the 50S 23S rRNA structure overlaid as yellow spheres (right). The helical regions not obscured by the yellow spheres are mostly disordered in the *Bbu* 50S density, suggesting displacement or internal conformation change in 23S RNA helices H68 and H69.

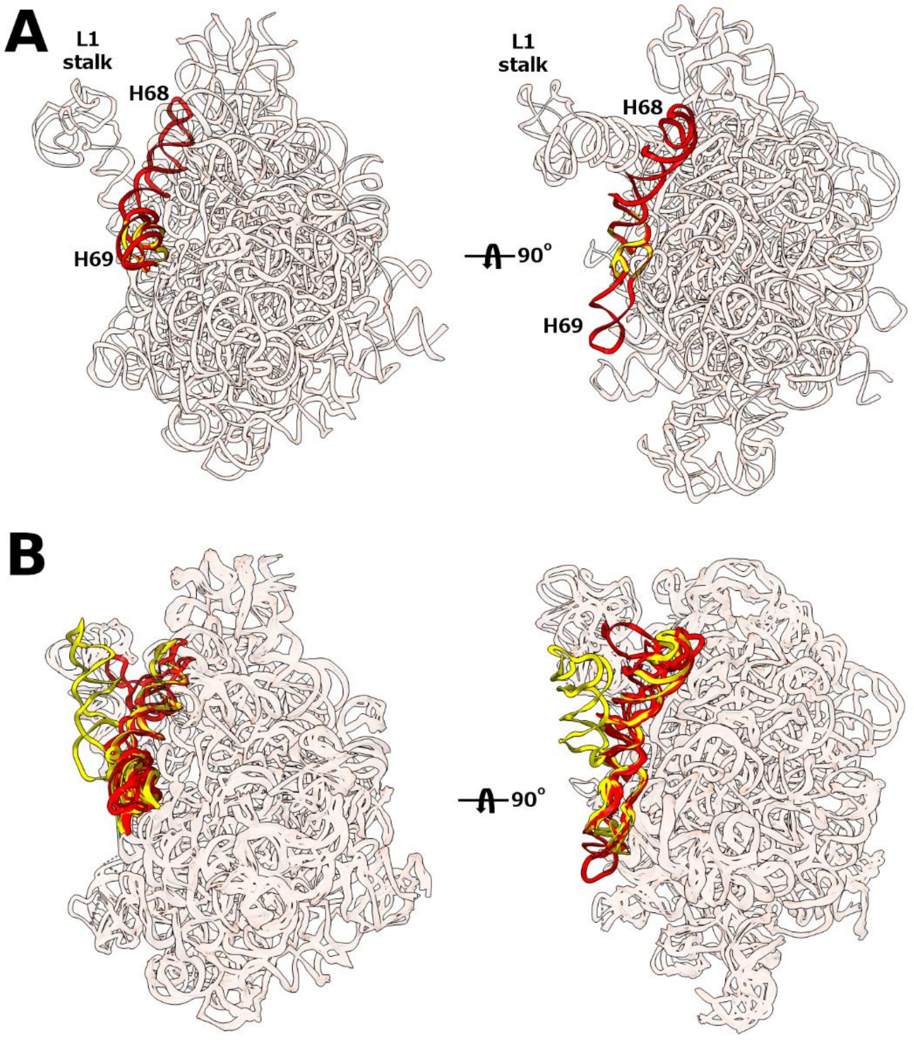

Supplementary Figure 13. Comparison of 23S RNA helix 68 (H68) and helix 69 (H69) in 70S and 50S in (A) *Bbu* and (B) *Staphylococcus aureus* (*Sau*). In panel B, the overlaid *Sau* structures for its 50S and 70S assemblies are shown for 3 durations of incubation at 37°C (in the format: PDB ID for 50S, PDB ID for 70S) as follows: 0 min (6HMA[53], 5TCU[54]), 30 min (7ASM[53], 7ASO[53]), 50 min (7ASN[53], 7ASP[53]). H68 and H69 in the 50S and 70S structures are shown in yellow and red, respectively.

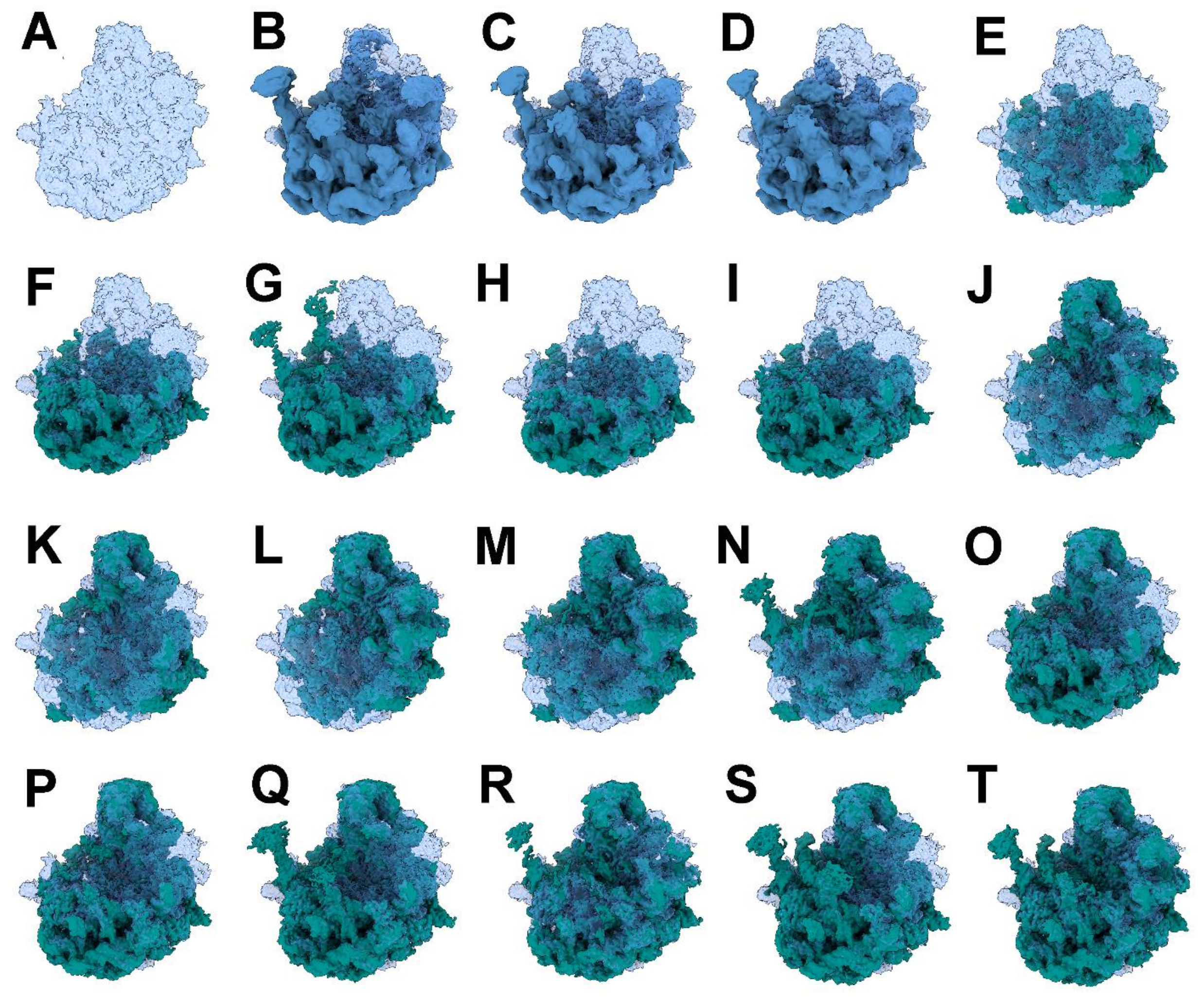

Supplementary Figure 14. Comparison of *Bbu* 50S subunit structure with 50S assembly intermediate structures from *Bacillus subtilis* (*Bsu*, in blue, B-D)[27] and *Eco* (in teal, E-T)[28]. **(A)** *Bbu* 50S subunit; Overlays of the *Bbu* 50S subunit with **(B)** 44.5S YsxC class I; **(C)** 44.5S YsxC class II; **(D)** 45S YphC; **(E)** Class B; **(F)** Class C; **(G)** Class C1; **(H)** Class C2; **(I)** Class C3; **(J)** Class D; **(K)** Class D1; **(L)** Class D2; **(M)** Class D3; **(N)** Class D4; **(O)** Class E; **(P)** Class E1; **(Q)** Class E2; **(R)** Class E3; **(S)** Class E4; **(T)** Class E5. Also see Supplementary Table 5.

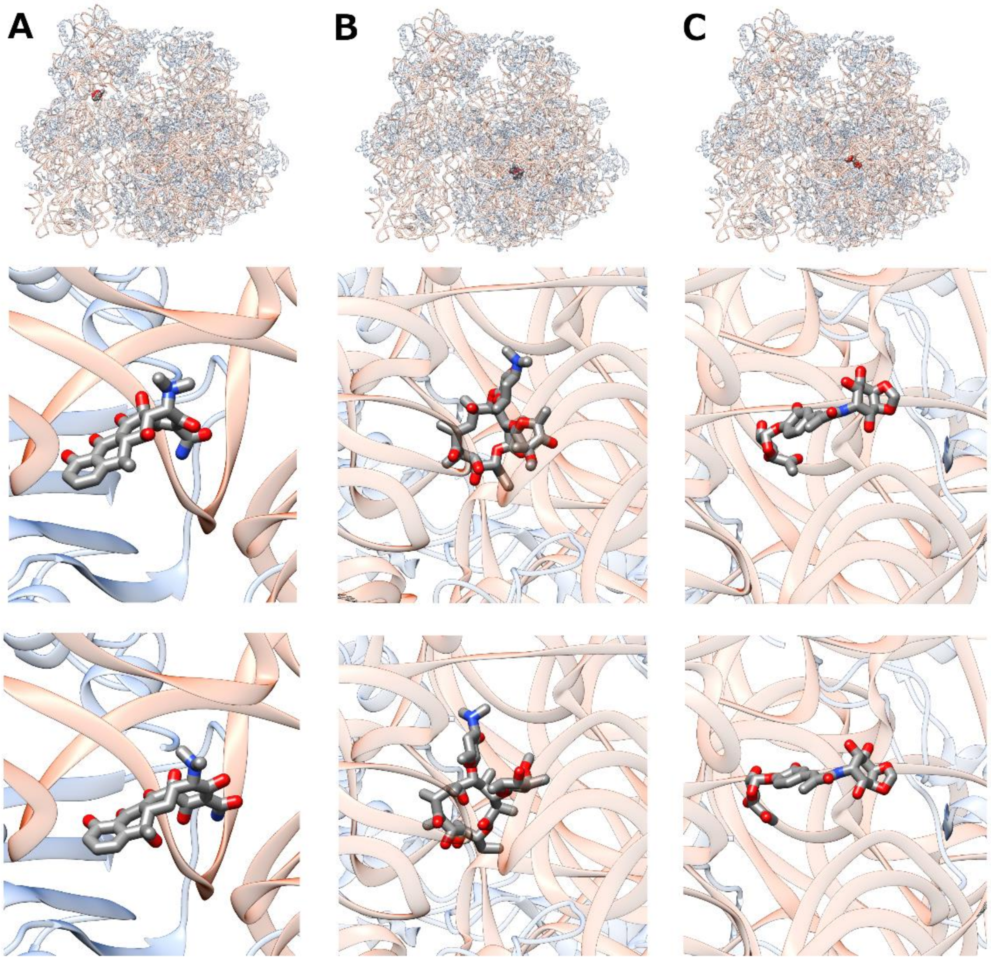

Supplementary Figure 15. Antibiotic binding to the *Bbu* 70S ribosome predicted using docking and structural analogy. (A) Doxycycline docked in the *Bbu* 70S ribosome (top), zoomed in view of docked doxycycline (middle), zoomed in view of tetracycline obtained by structural analogy (bottom, PDB ID: 5J5B[29]); **(B)** Erythromycin docked in the *Bbu* 70S ribosome (top), zoomed in view of docked erythromycin (middle), zoomed in view of erythromycin obtained by structural analogy (bottom, PDB ID: 6S0Z[12]); **(C)** Hygromycin A docked in the *Bbu* 70S ribosome (top), zoomed in view of docked hygromycin A (middle), zoomed in view of hygromycin A obtained by structural analogy (bottom, PDB ID: 5DM7[44]). Docking performed using Quickvina[55] with a box centered around the expected binding site, exhaustiveness parameter set to 32 and number of modes set to 100. Predicted Autodock Vina[56] binding free energies for the *Bbu* 70S ribosome docked positions of the antibiotics shown are as follows: doxycycline -5.4 kcal/mol, erythromycin -6.2 kcal/mol, hygromycin A -7.3 kcal/mol.

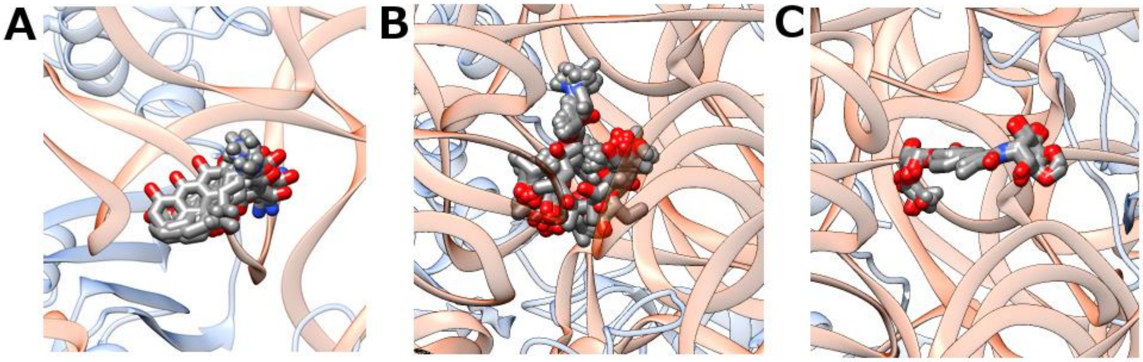

Supplementary Figure 16. Multiplicity in antibiotic binding conformations predicted using structural analogy. (A) Tetracycline in the small subunit decoding center; **(B)** Erythromycin near the peptidyl transferase center (PTC); **(C)** Hygromycin A near the PTC. The antibiotic structures are shown within the *Bbu* 70S ribosome. Structural analogy is obtained by a coarse alignment of the structures with ribosomal subunit RNA and then a finer alignment with neighboring residues within 10 Å of predicted bound antibiotic. Details for structures used in these overlays are in Table S4.

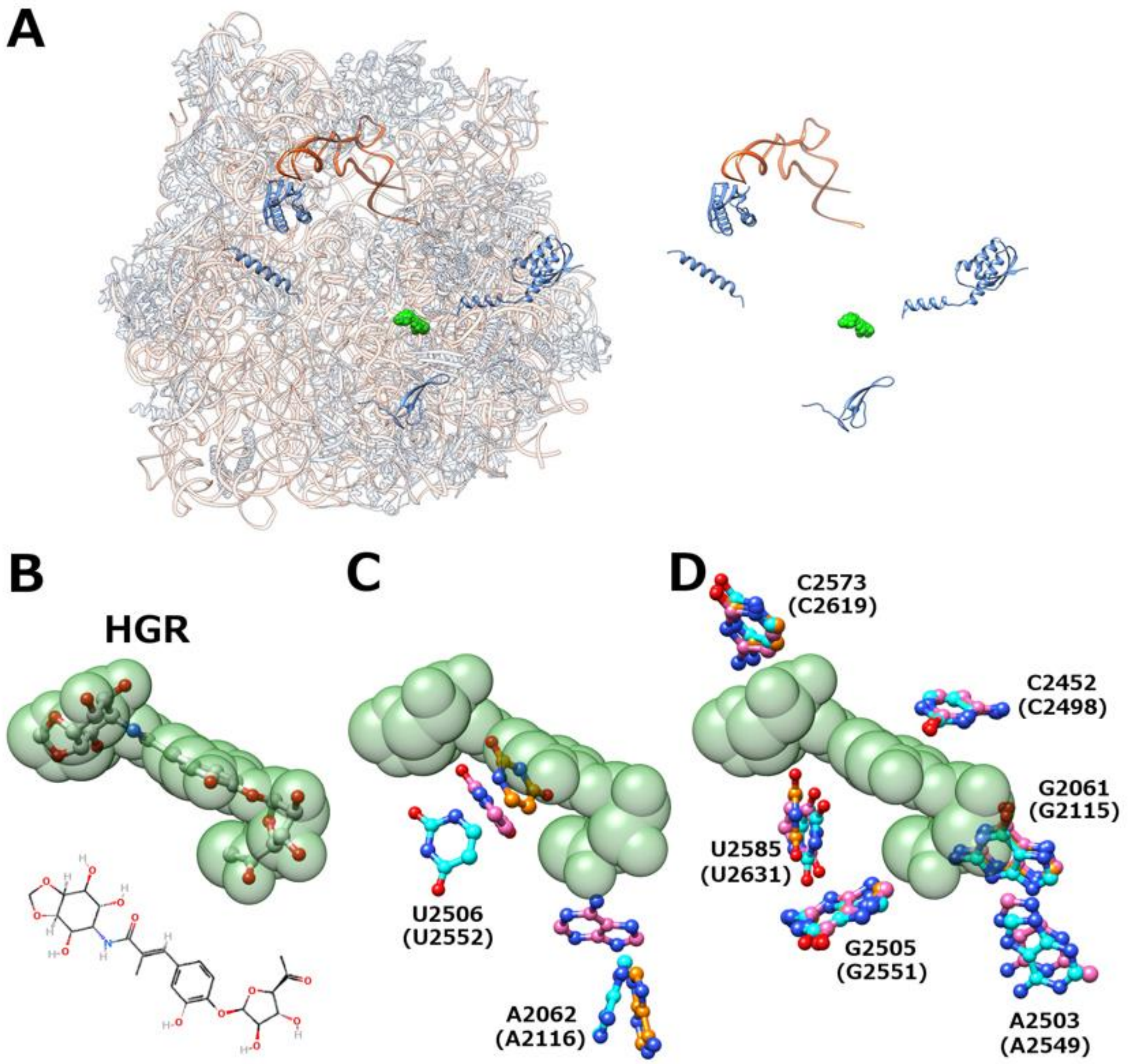

Supplementary Figure 17. Comparison of hygromycin A (HGR) binding pocket in *Bbu* and *Tth* ribosomes with and without bound HGR suggests that the empty *Bbu* HGR pocket may be more open for HGR accommodation. (A) Predicted HGR (green spheres) structure bound in the *Bbu* 50S subunit (left) with its distance from distinct proteins (blue ribbons) indicated (right); **(B)** HGR structure with transparent green spheres overlaid on its atoms (top) and its chemical structure (bottom); **(C)** HGR pocket 23S ribosomal RNA residues showing substantial variability between *Bbu* (this study), *Tth* (PDB ID: 4Y4O[11]), and *Tth* with HGR bound (PDB ID: 5DOX[43]) structures with changes in *Bbu* seemingly making more space for accommodation of HGR; (D) HGR pocket 23S ribosomal RNA residues showing substantial overlap between *Bbu*, *Tth*, and *Tth* HGR-bound structures. Base carbon atoms in 23S ribosomal RNA shown in cyan for *Bbu*, orange for *Tth*, and pink for *Tth* HGR-bound structures. *Bbu* numbering shown for 23S ribosomal RNA residues with *Tth* numbering in parentheses.

Supplementary References.

1 Hentschel, J. *et al.* The complete structure of the Mycobacterium smegmatis 70S ribosome. *Cell Reports* **20**, 149-160 (2017).

2 Li, Z. *et al.* Cryo-EM structure of Mycobacterium smegmatis ribosome reveals two unidentified ribosomal proteins close to the functional centers. *Protein & Cell* **9**, 384-388 (2018).

3 Li, Y. *et al.* Zinc depletion induces ribosome hibernation in mycobacteria. *Proceedings of the National Scademy of Sciences USA* **115**, 8191-8196, doi:10.1073/pnas.1804555115 (2018).

4 Mishra, S., Ahmed, T., Tyagi, A., Shi, J. & Bhushan, S. Structures of Mycobacterium smegmatis 70S ribosomes in complex with HPF, tmRNA, and P-tRNA. *Scientific Reports* **8**, 1-12 (2018).

5 Jha, V. *et al.* Structural basis of sequestration of the anti-Shine-Dalgarno sequence in the Bacteroidetes ribosome. *Nucleic Acids Research* **49**, 547-567 (2021).

6 Gertz, E. M., Yu, Y.-K., Agarwala, R., Schäffer, A. A. & Altschul, S. F. Composition-based statistics and translated nucleotide searches: improving the TBLASTN module of BLAST. *BMC Biology* **4**, 1-14 (2006).

7 Cao, X. & Slavoff, S. A. Non-AUG start codons: Expanding and regulating the small and alternative ORFeome. *Experimental Cell Research* **391**, 111973 (2020).

8 Villegas, A. & Kropinski, A. M. An analysis of initiation codon utilization in the Domain Bacteria–concerns about the quality of bacterial genome annotation. *Microbiology* **154**, 2559-2661 (2008).

9 Thompson, J. D., Gibson, T. J. & Higgins, D. G. Multiple sequence alignment using ClustalW and ClustalX. *Current Protocols in Bioinformatics*, 2.3. 1-2.3. 22 (2003).

10 De Bari, H. & Berry, E. A. Structure of Vibrio cholerae ribosome hibernation promoting factor. *Acta Crystallographica Section F: Structural Biology and Crystallization Communications* **69**, 228-236 (2013).

11 Polikanov, Y. S., Melnikov, S. V., Söll, D. & Steitz, T. A. Structural insights into the role of rRNA modifications in protein synthesis and ribosome assembly. *Nature Structural & Molecular Biology* **22**, 342-344 (2015).

12 Halfon, Y. *et al.* Exit tunnel modulation as resistance mechanism of S. aureus erythromycin resistant mutant. *Scientific Reports* **9**, 1-8 (2019).

13 Syroegin, E. A. *et al.* Structural basis for the context-specific action of the classic peptidyl transferase inhibitor chloramphenicol. *Nature Structural & Molecular Biology* **29**, 152-161 (2022).

14 Franklin, M. C. *et al.* Structural genomics for drug design against the pathogen Coxiella burnetii. *Proteins: Structure, Function, and Bioinformatics* **83**, 2124-2136 (2015).

15 Tereshchenkov, A. G. *et al.* Binding and action of amino acid analogs of chloramphenicol upon the bacterial ribosome. *Journal of Molecular Biology* **430**, 842-852 (2018).

16 Svetlov, M. S. *et al.* Structure of Erm-modified 70S ribosome reveals the mechanism of macrolide resistance. *Nature Chemical Biology* **17**, 412-420 (2021).

17 Polikanov, Y. S., Blaha, G. M. & Steitz, T. A. How hibernation factors RMF, HPF, and YfiA turn off protein synthesis. *Science* **336**, 915-918 (2012).

18 Seefeldt, A. C. *et al.* Structure of the mammalian antimicrobial peptide Bac7 (1–16) bound within the exit tunnel of a bacterial ribosome. *Nucleic Acids Research* **44**, 2429-2438 (2016).

19 Chen, C.-W. *et al.* Binding and action of triphenylphosphonium analog of chloramphenicol upon the bacterial ribosome. *Antibiotics* **10**, 390 (2021).

20 Matzov, D. *et al.* The cryo-EM structure of hibernating 100S ribosome dimer from pathogenic Staphylococcus aureus. *Nature Communications* **8**, 1-7 (2017).
21 Osterman, I. A. *et al.* Tetracenomycin X inhibits translation by binding within the ribosomal exit tunnel. *Nature Chemical Biology* **16**, 1071-1077 (2020).
22 Zhang, Z., Morgan, C. E., Bonomo, R. A. & Yu, E. W. Cryo-EM determination of Eravacycline-Bound structures of the Ribosome and the multidrug efflux pump AdeJ of Acinetobacter baumannii. *MBio* **12**, e01031-01021 (2021).
23 Beckert, B. *et al.* Structure of a hibernating 100S ribosome reveals an inactive conformation of the ribosomal protein S1. *Nature Microbiology* **3**, 1115-1121 (2018).
24 Mardirossian, M. *et al.* The dolphin proline-rich antimicrobial peptide Tur1A inhibits protein synthesis by targeting the bacterial ribosome. *Cell Chemical Biology* **25**, 530-539. e537 (2018).
25 Flyg rd, R. K., Boegholm, N., Yusupov, M. & Jenner, L. B. Cryo-EM structure of the hibernating Thermus thermophilus 100S ribosome reveals a protein-mediated dimerization mechanism. *Nature Communications* **9**, 1-12 (2018).
26 Li, Y. *et al.* Zinc depletion induces ribosome hibernation in mycobacteria. *Proceedings of the National Academy of Sciences USA* **115**, 8191-8196 (2018).
27 Ni, X. *et al.* YphC and YsxC GTPases assist the maturation of the central protuberance, GTPase associated region and functional core of the 50S ribosomal subunit. *Nucleic Acids Research* **44**, 8442-8455, doi:10.1093/nar/gkw678 (2016).
28 Davis, J. H. *et al.* Modular assembly of the bacterial large ribosomal subunit. *Cell* **167**, 1610-1622. e1615 (2016).
29 Cocozaki, A. I. *et al.* Resistance mutations generate divergent antibiotic susceptibility profiles against translation inhibitors. *Proceedings of the National Academy of Sciences USA* **113**, 8188-8193 (2016).
30 Jenner, L. *et al.* Structural basis for potent inhibitory activity of the antibiotic tigecycline during protein synthesis. *Proceedings of the National Academy of Sciences USA* **110**, 3812-3816 (2013).
31 Brodersen, D. E. *et al.* The structural basis for the action of the antibiotics tetracycline, pactamycin, and hygromycin B on the 30S ribosomal subunit. *Cell* **103**, 1143-1154 (2000).
32 Pioletti, M. *et al.* Crystal structures of complexes of the small ribosomal subunit with tetracycline, edeine and IF3. *The EMBO Journal* **20**, 1829-1839 (2001).
33 Tu, D., Blaha, G., Moore, P. B. & Steitz, T. A. Structures of MLSBK antibiotics bound to mutated large ribosomal subunits provide a structural explanation for resistance. *Cell* **121**, 257-270 (2005).
34 Svetlov, M. S. *et al.* High-resolution crystal structures of ribosome-bound chloramphenicol and erythromycin provide the ultimate basis for their competition. *RNA* **25**, 600-606 (2019).
35 Albers, S. *et al.* Repurposing tRNAs for nonsense suppression. *Nature Communications* **12**, 3850 (2021).
36 Beckert, B. *et al.* Structural and mechanistic basis for translation inhibition by macrolide and ketolide antibiotics. *Nature Communications* **12**, 4466 (2021).
37 Bulkley, D., Innis, C. A., Blaha, G. & Steitz, T. A. Revisiting the structures of several antibiotics bound to the bacterial ribosome. *Proceedings of the National Academy of Sciences USA* **107**, 17158-17163 (2010).
38 Wekselman, I. *et al.* The ribosomal protein uL22 modulates the shape of the protein exit tunnel. *Structure* **25**, 1233-1241. e1233 (2017).
39 Schlünzen, F. *et al.* Structural basis for the interaction of antibiotics with the peptidyl transferase centre in eubacteria. *Nature* **413**, 814-821 (2001).
40 Arenz, S. *et al.* A combined cryo-EM and molecular dynamics approach reveals the mechanism of ErmBL-mediated translation arrest. *Nature Communications* **7**, 12026 (2016).

41 Arenz, S. *et al.* Drug sensing by the ribosome induces translational arrest via active site perturbation. *Molecular Cell* **56**, 446-452 (2014).
42 Arenz, S. *et al.* Molecular basis for erythromycin-dependent ribosome stalling during translation of the ErmBL leader peptide. *Nature Communications* **5**, 3501 (2014).
43 Polikanov, Y. S. *et al.* Distinct tRNA accommodation intermediates observed on the ribosome with the antibiotics hygromycin A and A201A. *Molecular Cell* **58**, 832-844 (2015).
44 Kaminishi, T. *et al.* Crystallographic characterization of the ribosomal binding site and molecular mechanism of action of Hygromycin A. *Nucleic Acids Research* **43**, 10015-10025 (2015).
45 Pettersen, E. F. *et al.* UCSF ChimeraX: Structure visualization for researchers, educators, and developers. *Protein Science* **30**, 70-82 (2021).
46 Arenz, S., Nguyen, F., Beckmann, R. & Wilson, D. N. Cryo-EM structure of the tetracycline resistance protein TetM in complex with a translating ribosome at 3.9-Å resolution. *Proceedings of the National Academy of Sciences USA* **112**, 5401-5406 (2015).
47 Sprink, T. *et al.* Structures of ribosome-bound initiation factor 2 reveal the mechanism of subunit association. *Science Advances* **2**, e1501502 (2016).
48 Fischer, N. *et al.* Structure of the E. coli ribosome–EF-Tu complex at< 3 Å resolution by Cs-corrected cryo-EM. *Nature* **520**, 567-570 (2015).
49 Gagnon, M. G., Lin, J. & Steitz, T. A. Elongation factor 4 remodels the A-site tRNA on the ribosome. *Proceedings of the National Academy of Sciences USA* **113**, 4994-4999 (2016).
50 Fischer, N. *et al.* The pathway to GTPase activation of elongation factor SelB on the ribosome. *Nature* **540**, 80-85 (2016).
51 Nikolay, R. *et al.* Snapshots of native pre-50S ribosomes reveal a biogenesis factor network and evolutionary specialization. *Molecular Cell* **81**, 1200-1215. e1209 (2021).
52 Rundlet, E. J. *et al.* Structural basis of early translocation events on the ribosome. *Nature* **595**, 741-745 (2021).
53 Cimicata, G. *et al.* Structural Studies Reveal the Role of Helix 68 in the Elongation Step of Protein Biosynthesis. *MBio* **13**, e00306-00322 (2022).
54 Belousoff, M. J. *et al.* Structural basis for linezolid binding site rearrangement in the Staphylococcus aureus ribosome. *MBio* **8**, e00395-00317 (2017).
55 Alhossary, A., Handoko, S. D., Mu, Y. & Kwoh, C.-K. Fast, accurate, and reliable molecular docking with QuickVina 2. *Bioinformatics* **31**, 2214-2216 (2015).
56 Trott, O. & Olson, A. J. AutoDock Vina: improving the speed and accuracy of docking with a new scoring function, efficient optimization, and multithreading. *Journal of Computational Chemistry* **31**, 455-461 (2010).
