# Supplementary material for: The structure of a hibernating ribosome in a Lyme disease pathogen
Source: Nat Commun. 2023 Oct 31;14:6961. doi: 10.1038/s41467-023-42266-7 (PMC10618245; doi:10.1038/s41467-023-42266-7)
Supplement: Supplementary file 3 — Description of Additional Supplementary Files [file 41467_2023_42266_MOESM3_ESM.pdf]

File name: Supplementary Movie 1

Description: Movie showing features of the hibernating *Borrelia burgdorferi* (*Bbu*) 70S ribosome structure using a 360° rotation of the soft-lit cryo-EM density of the hibernating *Bbu* 70S ribosome in silver, a second 360° rotation with the cryo-EM density made transparent, a third 360° rotation with the RNA shown in orange-red ribbons, a fourth 360° rotation with the proteins also shown in cornflower-blue ribbons, and a fifth 360° rotation with only the following notable components shown in ribbons: E-tRNA in orange-red, bS22 in cyan, bL38 in green, uL30 in dark-green, and bbHPF in cornflower-blue.

File name: Supplementary Movie 2

Description: Movie showing relative motions between hibernation promotion factor (HPF) proteins and E-tRNA using three repetitions of transitions between HPF protein and E-tRNA relative orientations found in four hibernating ribosome structures in *Mycobacterium smegmatis* (*Msm*), *Escherichia coli* (*Eco*) and *Bbu*. For *Bbu*, protein is shown in cornflower- blue ribbons and E-tRNA in dark-red ribbons. For the other organisms, protein is shown in cyan and RNA in orange.

File name: Supplementary Movie 3

Description: Movie showing analogy between the uL30 protein in *Bbu* with large subunit ribosomal proteins in other organisms using a 360° rotation of the *Bbu* uL30 protein in cornflower-blue ribbons, a 360° rotation of the *Msm* uL30 protein in green ribbons and the bL37 protein in yellow ribbons, a 360° rotation of the *Homo sapiens* (*Hsa*) uL30 protein in orange ribbons, a 360° rotation of the *Hsa* mitochondrial uL30m protein in green ribbons and the mL63 protein in yellow ribbons, a 360° rotation of an overlay of the *Bbu* uL30 protein and the *Msm* uL30 and bL37 proteins, a 360° rotation of an overlay of the *Bbu* uL30 protein and the *Hsa* uL30 protein, and a 360° rotation of an overlay of the *Bbu* uL30 protein and the *Hsa* mitochondrial uL30m protein and the mL63 protein.

File name: Supplementary Movie 4

Description: Movie showing 23S RNA H68 and H69 disorder or motions using three repetitions of transitions between oriented 23S RNA structures found in eight ribosome structures from two bacteria: *Bbu* and *Staphylococcus aureus* (*Sau*), followed by their overlay. The structures are: *Bbu* 50S (PDB ID: 8FN2), *Bbu* 70S (PDB ID: 8FMW), *Sau* 50S with 0 minutes incubation at 37 °C (PDB ID: 6HMA), *Sau* 50S with 30 minutes incubation at 37 °C (PDB ID: 7ASM), *Sau* 50S with 50 minutes incubation at 37 °C (PDB ID: 7ASN), *Sau* 70S with 0 minutes incubation at 37 °C (PDB ID: 5TCU), *Sau* 70S with 30 minutes incubation at 37 °C (PDB ID: 7ASO), *Sau* 70S with 50 minutes incubation at 37 °C (PDB ID: 7ASP). For *Bbu*, 23S RNA residues 1888-2001 are shown in green, for *Sau* corresponding residues 1861-1974 are shown in yellow.
