# Supplementary material for: The structure of a hibernating ribosome in a Lyme disease pathogen
Source: Nat Commun. 2023 Oct 31;14:6961. doi: 10.1038/s41467-023-42266-7 (PMC10618245; doi:10.1038/s41467-023-42266-7)
Supplement: Supplementary file 8 — Reporting Summary [file 41467_2023_42266_MOESM8_ESM.pdf]

nature portfolio

Corresponding author(s): Nilesh K. Banavali, Rajendra K. Agrawal

Last updated by author(s): Sep 29, 2023

Reporting Summary

Nature Portfolio wishes to improve the reproducibility of the work that we publish. This form provides structure for consistency and transparency in reporting. For further information on Nature Portfolio policies, see our Editorial Policies and the Editorial Policy Checklist.

Statistics

For all statistical analyses, confirm that the following items are present in the figure legend, table legend, main text, or Methods section.

n/a	Confirmed	
☐	☒	The exact sample size (*n*) for each experimental group/condition, given as a discrete number and unit of measurement
☐	☒	A statement on whether measurements were taken from distinct samples or whether the same sample was measured repeatedly
☒	☐	The statistical test(s) used AND whether they are one- or two-sided<br>*Only common tests should be described solely by name; describe more complex techniques in the Methods section.*
☒	☐	A description of all covariates tested
☒	☐	A description of any assumptions or corrections, such as tests of normality and adjustment for multiple comparisons
☒	☐	A full description of the statistical parameters including central tendency (e.g. means) or other basic estimates (e.g. regression coefficient) AND variation (e.g. standard deviation) or associated estimates of uncertainty (e.g. confidence intervals)
☒	☐	For null hypothesis testing, the test statistic (e.g. *F*, *t*, *r*) with confidence intervals, effect sizes, degrees of freedom and *P* value noted<br>*Give P values as exact values whenever suitable.*
☒	☐	For Bayesian analysis, information on the choice of priors and Markov chain Monte Carlo settings
☒	☐	For hierarchical and complex designs, identification of the appropriate level for tests and full reporting of outcomes
☒	☐	Estimates of effect sizes (e.g. Cohen's *d*, Pearson's *r*), indicating how they were calculated

Our web collection on statistics for biologists contains articles on many of the points above.

Software and code

Policy information about availability of computer code

Data collection	Appion (Lander GC, Stagg SM, Voss NR, Cheng A, Fellmann D, Pulokas J, Yoshioka C, Irving C, Mulder A, Lau PW, Lyumkis D, Potter CS, Carragher B. Appion: an integrated, database-driven pipeline to facilitate EM image processing. J Struct Biol. 2009 Apr;166(1):95-102. doi: 10.1016/j.jsb.2009.01.002. PMID: 19263523; PMCID: PMC2775544.)<br>Leginon (Carragher B, Kisseberth N, Kriegman D, Milligan RA, Potter CS, Pulokas J, Reilein A. Leginon: an automated system for acquisition of images from vitreous ice specimens. J Struct Biol. 2000 Oct;132(1):33-45. doi: 10.1006/jsbi.2000.4314. PMID: 11121305.)
Data analysis	cryoSPARC v2.15 (Punjani A, Rubinstein JL, Fleet DJ, Brubaker MA. cryoSPARC: algorithms for rapid unsupervised cryo-EM structure determination. Nat Methods. 2017 Mar;14(3):290-296. doi: 10.1038/nmeth.4169. Epub 2017 Feb 6. PMID: 28165473.)<br>SerialEM (Schorb M, Haberbosch I, Hagen WJH, Schwab Y, Mastronarde DN. Software tools for automated transmission electron microscopy. Nat Methods. 2019 Jun;16(6):471-477. doi: 10.1038/s41592-019-0396-9. Epub 2019 May 13. PMID: 31086343; PMCID: PMC7000238.)<br>RNA PDBEE (Zok T, Antczak M, Zurkowski M, Popenda M, Blazewicz J, Adamiak RW, Szachniuk M. RNApdbee 2.0: multifunctional tool for RNA structure annotation. Nucleic Acids Res. 2018 Jul 2;46(W1):W30-W35. doi: 10.1093/nar/gky314. PMID: 29718468; PMCID: PMC6031003.)<br>R2DT (Sweeney BA, Hoksza D, Nawrocki EP, Ribas CE, Madeira F, Cannone JJ, Gutell R, Maddala A, Meade CD, Williams LD, Petrov AS, Chan PP, Lowe TM, Finn RD, Petrov AI. R2DT is a framework for predicting and visualising RNA secondary structure using templates. Nat Commun. 2021 Jun 9;12(1):3494. doi: 10.1038/s41467-021-23555-5. PMID: 34108470; PMCID: PMC8190129.)<br>Alphafold2 (Jumper J, Evans R, Pritzel A, Green T, Figurnov M, Ronneberger O, Tunyasuvunakool K, Bates R, Žídek A, Potapenko A, Bridgland A, Meyer C, Kohl SAA, Ballard AJ, Cowie A, Romera-Paredes B, Nikolov S, Jain R, Adler J, Back T, Petersen S, Reiman D, Clancy E, Zielinski M, Steinegger M, Pacholska M, Berghammer T, Bodenstein S, Silver D, Vinyals O, Senior AW, Kavukcuoglu K, Kohli P, Hassabis D. Highly accurate protein structure prediction with AlphaFold. Nature. 2021 Aug;596(7873):583-589. doi: 10.1038/s41586-021-03819-2. Epub 2021 Jul 15. PMID: 34265844; PMCID: PMC8371605.)<br>UCSF Chimera v1.16 (Pettersen EF, Goddard TD, Huang CC, Couch GS, Greenblatt DM, Meng EC, Ferrin TE. UCSF Chimera--a visualization

system for exploratory research and analysis. J Comput Chem. 2004 Oct;25(13):1605-12. doi: 10.1002/jcc.20084. PMID: 15264254.)
UCSF ChimeraX 1.4 (Pettersen EF, Goddard TD, Huang CC, Meng EC, Couch GS, Croll TI, Morris JH, Ferrin TE. UCSF ChimeraX: Structure visualization for researchers, educators, and developers. Protein Sci. 2021 Jan;30(1):70-82. doi: 10.1002/pro.3943. Epub 2020 Oct 22. PMID: 32881101; PMCID: PMC7737788.)
Inkscape (Bah T. Inkscape: guide to a vector drawing program. Upper Saddle River: Prentice Hall; 2011 May 6.)
Gimp 2.10 (Peck A. Beginning GIMP: from novice to professional. Apress; 2006 Nov 21.)
Scaffold5 (https://www.proteomesoftware.com/products/scaffold-5)

For manuscripts utilizing custom algorithms or software that are central to the research but not yet described in published literature, software must be made available to editors and reviewers. We strongly encourage code deposition in a community repository (e.g. GitHub). See the Nature Portfolio guidelines for submitting code & software for further information.

Data

Policy information about availability of data

All manuscripts must include a data availability statement. This statement should provide the following information, where applicable:

- Accession codes, unique identifiers, or web links for publicly available datasets
- A description of any restrictions on data availability
- For clinical datasets or third party data, please ensure that the statement adheres to our policy

The data generated in this study are available in the following databases:

8FMW [http://doi.org/10.2210/pdb8fmw/pdb] (Borrelia burgdorferi hibernating 70S ribosome)
8FN2 [http://doi.org/10.2210/pdb8fn2/pdb] (Borrelia burgdorferi 50S subunit)

EMD-29298 [https://www.ebi.ac.uk/emdb/EMD-29298] (Borrelia burgdorferi hibernating 70S ribosome)
EMD-29304 [https://www.ebi.ac.uk/emdb/EMD-29304] (Borrelia burgdorferi 50S subunit)

These data are only held until publication, there are no restrictions on data availability after publication.

Research involving human participants, their data, or biological material

Policy information about studies with human participants or human data. See also policy information about sex, gender (identity/presentation), and sexual orientation and race, ethnicity and racism.

Reporting on sex and gender	Not applicable
Reporting on race, ethnicity, or other socially relevant groupings	Not applicable
Population characteristics	Not applicable
Recruitment	Not applicable
Ethics oversight	Not applicable

Note that full information on the approval of the study protocol must also be provided in the manuscript.

Field-specific reporting

Please select the one below that is the best fit for your research. If you are not sure, read the appropriate sections before making your selection.

☒ Life sciences ☐ Behavioural & social sciences ☐ Ecological, evolutionary & environmental sciences

For a reference copy of the document with all sections, see nature.com/documents/nr-reporting-summary-flat.pdf

Life sciences study design

All studies must disclose on these points even when the disclosure is negative.

Sample size	Not relevant to this study.
Data exclusions	No data were excluded.
Replication	No findings were replicated.
Randomization	Not relevant to this study.
Blinding	Not relevant to this study.

Reporting for specific materials, systems and methods

We require information from authors about some types of materials, experimental systems and methods used in many studies. Here, indicate whether each material, system or method listed is relevant to your study. If you are not sure if a list item applies to your research, read the appropriate section before selecting a response.

Materials & experimental systems

n/a	Involved in the study
☒	☐ Antibodies
☒	☐ Eukaryotic cell lines
☒	☐ Palaeontology and archaeology
☒	☐ Animals and other organisms
☒	☐ Clinical data
☒	☐ Dual use research of concern
☒	☐ Plants

Methods

n/a	Involved in the study
☒	☐ ChIP-seq
☒	☐ Flow cytometry
☒	☐ MRI-based neuroimaging
